# Epidemiology and risk factors of surgical site infections in elective surgeries in Pakistan (2022–2023): a multicentre, prospective cohort study from PakSurg 1

**DOI:** 10.1016/j.lansea.2026.100786

**Published:** 2026-06-01

**Authors:** Usama Waqar, Shaheer Ahmed, Asad Saulat Fatimi, Warda Ahmed, Russell Seth Martins, Haseeb Waheed, Illiyun Banani, Dahir Ashfaq, Asma Altaf Merchant, Ronika Devi Ukrani, Manzar Abbas, Muskan Abdul Qadir, Izza Tahir, Daniyal Ali Khan, Hareem Rauf, Mahnoor Javaid, Sarim Raheel, Muhammad Uzair, Muhammad Umar Mahar, Sehar Salim Virani, Faiqa Binte Aamir, Hamna Ganny, Mabrooka Kazi, Hajra Arshad, Muhammad Abbas Raza, Muhammad Jawad Amin Malik, Muhammad Ozair Awan, Abida Khalil Sattar, Amna Urooba, Aliya Begum Aziz, Erum Baig, Maheen Mansoor, Hina Inam, Mashal Shah, Muhammad Shahzad Shamim, Nadeem Siddiqui, Rehana Siddiqui, Sadaf Khan, Shahryar Noordin, Tabish Chawla, Syed Ather Enam, PakSurg Collaborative, Usama Waqar, Shaheer Ahmed, Asad Saulat Fatimi, Warda Ahmed, Russell Seth Martins, Haseeb Waheed, Illiyun Banani, Dahir Ashfaq, Asma Altaf Merchant, Ronika Devi Ukrani, Manzar Abbas, Muskan Abdul Qadir, Izza Tahir, Daniyal Ali Khan, Hareem Rauf, Mahnoor Javaid, Sarim Raheel, Muhammad Uzair, Muhammad Umar Mahar, Sehar Salim Virani, Faiqa Binte Aamir, Hamna Ganny, Mabrooka Kazi, Hajra Arshad, Muhammad Abbas Raza, Muhammad Jawad Amin Malik, Muhammad Ozair Awan, Abida Khalil Sattar, Amna Urooba, Aliya Begum Aziz, Erum Baig, Maheen Mansoor, Hina Inam, Mashal Shah, Muhammad Shahzad Shamim, Nadeem Siddiqui, Rehana Siddiqui, Sadaf Khan, Shahryar Noordin, Tabish Chawla, Syed Ather Enam, Laiba Kiani, Naeem Ahmed, Shafaq Hanif, Mahnoor Zahra, Amna Aslam, Haasin Iqbal, Zarish Shuja, Huzaifa Shabbir, Umm-e-Roman, Areeba Kabeer, Amerzish Shahid, Aqsa Haider, Fatima Tanveer, Marriam Nazakat, Alveena Waheed, Sana Umer Kiani, Mehrish Batool, Sana Kokab, Syed Nazar Sherazi, Mifrah Rahat Khan Sherwani, Gulnaz Khalid, Muhammad Tahir, Aisha Khatoon, Zainab Farooq, Shanza Gul, Hira Islam, Areeba Saleem, Aariz Hussain, Wajiha Shaikh, Areeba Fareed, Warisha Kanwal, Vousqa Zubair Ahmed, Aqsa Fareed, Fareeha, Sana Iqbal, Zoha Haq, Kanwar Arham, Abdul Haseeb, Anum Khalid, Iqra, Tazmeen Sabooh, Hania Arshad, Faiza Saleem, Tehreem, Fariya Majid, Sibqun Irfan, Abdul Ghafoor Qureshi, Moaaz Syed Nezami, Farhan Ahmed, Madeeha Ali, Muhammad Taha Nasim, Umer Adnan, Bilal Lodhi, Muhammad Tabish Nasim, Linta Khan, Khushi Saleem, Muneeb Khalid, Mannal Ahmed, Muhammad Ibrahim, Maryam Shaukat, Faiza Qureshi, Sneha Bheesham, Zain Javed, Taha Shaikh, Syeda Samnita Batool Zaidi, Aqsa Amjad, Huzaifa Ahmed, Fatima Abdullah, Fiza Adnan Khan, Sibgha Alam, Konain Imran, Reyan Hussain Shaikh, Muneeb Ahmed, Saba Bilal Qamar, Abdul Hadi Shahid, Sana Farhan, Hania Fatima, Arshia Jahangir, Umme Abeeha Zafar, Wamiq Ali Shaikh, Muhammad Hyedar Anwar, Muhammad Abdullah Jamil, Hashim Ishfaq, Hufriya Mondegarian, Mohammad Zakriya, Varisha Madni, Mohammad Shahmeer Chaudhry, Hamza Haider, Hunaina Abid, Komal Fida Ali, Ismail Khan, Khadija Awais Sumra, Amna Irfan Ansari, Misbah Jahangir, Aliha Shabbir, Sheza Saqib, Ahmad Jan Anab, Eisha Saadat, Aiman Sultan, Yusra Imran, Talal Bin Tariq, Kinza Jawed, Umair Saleem, Kashmala Hussain, Musa Salar, Hashim Salar, Hassan Khan Niazi, Sajjan Raja, Zayan Alidina, Mashal Waqas, Fiza Sohail Gagai, Abia Abdullah, Shiza Atif, Karishma Wali, Zainab Haider Ejaz, Maha Muzzamil Chaipiwala, Shilpa Golani, Alizeh Sonia Fatimi, Shalni Golani, Abdullah Ahmed, Khadijah Aslam, Maheen Qureshi, Muhammad Daniyal, Eman Anwar, Shahier Paracha, Salaar Ahmed, Shahzil Abdur Rehman Malik, Ayesha Nawal, Muhammad Qasim Butt, Muhammad Luqman Kaleem, Abdur Rehman, Nabiha Syed, Muhammad Waleed Ajmal, Unaiza Ahmad, Muhammad Saleem Iqbal, Nazar Hussain, Ammara Niaz, Muhammad Shoaib Bin Shakeel, Azka Irfan, Mahrukh Atif, Mahnoor Naeem, Noor Azhar, Labeeba Abdul Ghafoor, Momna Yousaf, Muhammad Bilal Ashraf, Ajia Ali Khan, Sumayya Sajid, Muhammad Ahmad Arsal, Abdul Moeid, Abdullah Nasir, Mashal Fatima Farooq, Syed Samar Ali Shah, Ahmad Hassan Gul, Irtaza Shafqat, Hafiz Muhammad Usama Zuhair, Umer Hussain, Muhammad Ali, Sarmad Naeem, Ahmad Butt, Kiran Fatima, Rameen Fatima, Maryam Haroon, Muhammad Usman Javed, Muhammad Huzaifa Khan, Muhammad Awais Ali, Sameer Ashraf, Bilal Habib, Ali Shehzad, Ammara Basit, Nahal Irshad, Abrar Ahmad, Ayyaz Ahmad, Syed Sheraz Ashiq, Mubeen Ahmad Randhawa, Adnan Ansar Sukhyra, Musharraf Ur Rehman, Ahsan Ali Akbar, Muhammad Zain Munir, Ghazi Umair Ahmad, Muhammad Zaman, Shah Ahmed Cheema, Nadeem Ahmed, Muhammad Iltaf, Ruqqia Sultana, Fazli Junaid, Wajeeha Khurshid, Muhammad Qaisar Shah, Jamal Ali, Sakhawat Ali, Salman Ahmad Khan, Muhammad Raza, Zubair Ahmed, Shakeel Akbar, Saiqa Bazai, Abdifitah Bashir Omer, Fouzia Ali, Hunniya Bint-e-Riaz, Muhammad Akmal, Muhammad Imran, Ismail Mazhar, Mir Muhammad Rai, Rao Muhammad Waleed, Maaela Khan, Amir Iqbal Ali, Maryam Zubair, Arooj Sabahat, Talha Adil, Zoha Khan, Rehnaz Riaz, Poshmal Zahid, Arwah Asif, Muhammad Qaisar Karim, Afroza Abbas, Ahmad Hassan Khan, Sufian Muhammad Zahid, Muhammad Muneer Haider, Muhammad Hassan Javeed, Areeba Mariam Mahmood, Muhammad Arsalan, Nabeel Akhtar, Saad Maqbool, Ahmed Raza, Munazzah Aziz, Muhammad Assad Javed, Bushra Kant, Muhammad Hanif, Muhammad Khurram Sajjad, Sarah Ishtiyaq, Zainab Shahid, Sidra Javaid, Areej Taswar, Fatima Batool, Syeda Minahal Kazmi, Sana Razzaq, Uroba Arshad, Amal Khan, Mahad Ali Khan, Arrham Hai, Syeda Zohva Zainub, Maham Farooq, Shehryar Javed, Muhammad Asher Javed, Anam Zafar, Eisha Faheem, Saliha Maqsood, Shaiza Jabbar, Noor Ul Zuha, Soma Siddique, Zubaria Qureshi, Muhammad Ali, Izah Sadiq, Amna Shahid, Rimsha Zahid, Khaleeq Ur Rehman, Javed Shakir, Andleeb Kanwal, Noor Fatima, Hamna Zaman, Amun Mustafa, Mohammad Ahmad Farooq, Fizza Mansoor, Irtiqua Zaheer, Maham Murtaza, Sameen Fatima Zafar, Hassan Sarwar, Muhammad Umair Amjad, Laila Khadim, Zahid Aman, Muhammad Aasim, Muhammad Waqar, Iqra Zaman, Muhammad Sheraz Ali, Mahnoor Musharaf, Hafsa Khan, Sadia Ambreen, Aqdas Faiz, Aiman Haroon, Mehreen Mushtaq Ahmad, Alishba Fatima, Maham Mehmood, Eeman Shehzad, Iman Ali, Shandana Gul, Maimoona Naeem, Shanza Abbasi, Jahangir Sarwar Khan, Rubina Shahzad, Gohar Rasheed, Ashraf, Usman Qureshi, Lubna Ijaz, Zubair, Fatima Siddiqui, Juwereya Memon, Farhana Anjum, Mohammad Shahid Khan, Adeena Noor, Fiza Lashari, Alizeh Fatima Memon, Mariam Qureshi, Amna Gopang, Aisha Shah, Marvi Mirbhar, Anousha Afzal, Abdullah Memon, Seerat Hameem, Sadia Hameed Lashary, Khushboo Devi, Adeel Rehman, Ahmed Mustafa Burney, Muhammad Alyaan Arshad, Shahid Mehmood, Muhammad Umer Farooq, Raffay Ali Gillani, Nabeel Chodhary, Saima Iqbal, Laveeza Fatima, Minahil Iqbal, Faseeh Fatima, Sara Khan, Anzil Adnan, Farooq Ahmad, Rubab Zahra, Sehar Fatima, Muhammad Fazeel Shahid, Muhammad Zulkifl, Hadia Nadeem, Muhammad Subhan Saleem, Hassan Mehmood, Muhammad Ahrar Bin Naeem, Tooba Nihal, Ayesha Yousaf Khan, Yumna Naeem, Aqsa Zainab, Laiba Ali, Saad Masood, Ali Azlan, Abdul Wasay, Abdul Wahab Mirza, Marium Mansoor, Maryam Baloch, Zain Ali Nadeem, Maha Malik, Muhammad Rafay Paracha, Muhammad Hamza Awais Khalid, Hammad Jehangir, Yashfeen Amjad, Shahab Zafar, Sameen Shafqat, Haider Ashfaq, Hamza Ashraf, Sophia Ahmed, Aaila Hameed, Areej Iftikhar, Asna Moghis, Umar Akram, Faryal Khan, Samra Shafique, Muhammad Sohaib Khan, Fasih Khalil Ur Rehman, Abdullah Arshad, Ali Nawaz Khan, Muhammad Zareen, Muhammad Idris Khan, Fauzia Afridi, Sadaf Faryal, Arif Hussain, Romana Bibi, Sara Jamil, Waseem Jamil, Sundas Mehreen, Abdul Wahab, Haseena Rehman, Saima Khattak, Faaiz Ali Shah, Ilyas Khan, Ijaz Ahmad, Kainat Ullah Khan, Ume Kulsum, Tariq Shah, Abdullah Khan, Babur Farid, Abbas Bangash, Syed Mubeen Ahmed, Ahmed Naeem, Yaser Ul Din Hoti, Farooq Afzal, Adeel Hamid, Najam Gohar, Mahin Qudeer Sheikh, Umar Abdullah, Waleed Anjum, Uzair Ahmad, Abdullah Masood, Aroob Farooqi, Suman Memon, Vashdev Khimani, Ahmed Hussain Pathan, Qurat Ul Ain, Fahad Shabir, Taskeen Waheed, Usama Ahmed Qureshi, Ramsha Anwar, Nadia Sadiq Memon, Sanjeet Kumar, Farhat Bano, Javeria Farooq, Faisal Aftab, Fehmina Raza, Muhammad Shaharyar Sabeeh, Nouman Arif, Shams Bukhari, Aamir Shahzad, Rizwan Sharif, Munazza Khalid, Muhammad Ali, Ali Waqas, Syed Fouz Hyder Abidi, Muhammad Uzair Rafique, Musfirah Younis, Wajeeha Younas, Rimsha Afzal, Laraib Mubashir, Abdul Rafay, Aqsa Bilal, Naima Khalid, Mohammad Nouman Arshad, Almas Fasih Khattak, Tarbia Hamid, Syed Sarmad Bukhari, Amjad Ali Shah, Saifullah Shafiq, Muhammad Hamza, Fatima Khan, Aamena Akhtar, Kiran Rehman, Ayesha Mobeen, Masooma Husnain, Zainab Zia, Iqra Nasir, Mohad Bin Asghar, Syed Usman Husain Shah, Zobia Majeed, Lareb Basharat, Sumaira Yasmeen, Raja Adnan, Rehan Ahmed Khan, Sohail Iqbal Sheikh, Sameen Rehan, Sobul Khan, Mohammad Ali Sohail, Naeem Karim Bhatti, Abdul Razaque Mari, Manahil Iftekhar, Sanish Abassi, Wardah Nizamani, Amna Suhail, Mahek Gul, Summiya Bhatti, Kiran Manwani, Haseeb Mehmood Qadri, Asif Bashir, Areesha Fazil Awan, Rai Muhammad Aslam, Tabassum Firdous

**Affiliations:** Aga Khan University Hospital, Karachi, Pakistan

**Keywords:** Surgical site infections, Surgical wound infection, Postoperative complications, Incidence, Risk factors, Disease burden, Prevention

## Abstract

**Background:**

Surgical site infections (SSIs) are a leading and preventable cause of postoperative morbidity in Pakistan. However, the absence of national-level data has precluded the development of targeted preventative measures. PakSurg1 aimed to quantify the burden of SSIs and evaluate associated risk factors in a multicentre cohort across Pakistan.

**Methods:**

We conducted a prospective cohort study of 1711 patients across 28 hospitals in Pakistan from 2022 to 2023. Adult patients undergoing elective general or subspecialty surgery were followed for 30 days postoperatively. We assessed SSI incidence and identified risk factors at the patient, surgical, and hospital levels.

**Findings:**

The overall incidence of SSIs was 9.5% (n = 163). Most SSIs were superficial (73.6%; n = 120) and managed non-surgically with antibiotics (66.9%; n = 109). On multivariable analysis, SSIs were more likely in patients undergoing trauma-related surgery (OR: 3.047 [95% CI: 1.205–7.708]), with contaminated or dirty wounds (OR: 3.245 [95% CI: 1.298–8.113]), or with longer hospital stays (OR: 1.068 [95% CI: 1.036–1.101]). Several modifiable factors were associated with lower odds of SSI. These included laparoscopic surgery (OR: 0.556 [95% CI: 0.343–0.900]), ambulatory procedures (OR: 0.598 [95% CI: 0.375–0.953]), combined chlorhexidine and povidone-iodine skin preparation (OR: 0.304 [95% CI: 0.131–0.704]), and completion of a surgical time-out (OR: 0.446 [95% CI: 0.268–0.743]). Compared to general surgery, lower SSI odds were observed in cardiac (OR: 0.223 [95% CI: 0.074–0.672]), cranio-spinal (OR: 0.292 [95% CI: 0.126–0.676]), and orthopaedic (OR: 0.216 [95% CI: 0.071–0.655]) procedures.

**Interpretation:**

PakSurg 1 provides a national estimate of SSI incidence in elective surgeries in Pakistan and identifies key, modifiable risk factors. These findings can inform targeted, context-specific strategies to reduce SSIs and improve surgical safety nationwide.

**Funding:**

This study received monetary funding from the Student & Trainee Initiated Research Program (MBBS Class of ’88 Awards, Aga Khan University, Karachi, Pakistan).

**Research in context**

**Evidence before this study**

We searched PubMed, Google Scholar, and local databases for studies on surgical site infections (SSIs) in Pakistan published up to March 2026, using terms such as “surgical site infection,” “wound infection,” “LMIC,” and “Pakistan,” without language restrictions. Most identified studies were small, single-centre, and retrospective, reporting SSI rates between 9% and 34%. Methodological limitations were common, including inconsistent definitions, limited follow-up, and inadequate risk adjustment. No study provided nationally representative data or comprehensively examined modifiable perioperative practices, highlighting the need for a large, prospective, multicentre study.

**Added value of this study**

PakSurg1 is one of the first nationally representative, prospective cohort study to evaluate the burden and risk factors of SSIs across Pakistan. It included 1711 patients from 28 hospitals, capturing a wide range of geographic and clinical settings. Using standardized CDC criteria, active follow-up, and multivariable analysis, this study identified key modifiable risk factors for SSIs and provides robust evidence to inform targeted infection prevention strategies.

**Implications of all the available evidence**

Our findings, when combined with existing evidence, highlight the urgent need for standardized SSI prevention strategies in Pakistan. The study supports revising national surgical guidelines to include dual-agent skin preparation and mandatory time-outs, expanding surveillance in high-risk specialties such as trauma and colorectal surgery, and strengthening laparoscopic infrastructure to reduce infection risk. Future research should evaluate cost-effective interventions suited to resource-limited settings, where even incremental improvements could yield significant reductions in SSI rates and their associated health and economic burdens.

## Introduction

Surgical site infections (SSIs) are among the most prevalent and debilitating postoperative complications, posing a significant threat to patient recovery and surgical outcomes worldwide.^1^^,^^2^ Negative clinical outcomes associated with SSIs include prolonged hospital stays, increased postoperative morbidity and mortality, reoperation, and reduced quality of life.^3^^,^^4^ Inevitably, SSIs also impose significant financial strain on already vulnerable individuals, with some studies suggesting that the incidence of an SSI may increase the healthcare-associated costs of patients by up to 226%.^5^^,^^6^ In low- and middle-income countries (LMICs) such as Pakistan where out-of-pocket expenses dominate healthcare financing,^7^ SSIs are a major contributor to catastrophic healthcare expenditures, pushing families into poverty.^6^^,^^8^^,^^9^

Despite these devastating impacts and a disproportionately higher incidence of SSIs in LMICs compared to high-income countries (HICs),^10^^,^^11^ data-driven approaches in LMICs to prevent SSIs are sorely lacking.^4^ Significant challenges, including inadequate infrastructure and limited human resources, hinder the reliable data collection essential for driving such approaches in LMICs.^12^ In Pakistan, the literature on SSIs remains limited to single-centre retrospective studies which lack the standardization necessary for national generalizability and are subject to significant bias and confounding.^13^, ^14^, ^15^, ^16^, ^17^ As a result, accurate assessment of SSI incidence and risk factors, as well as the successful implementation of effective, context-specific preventive measures, remains a substantial challenge in Pakistan.

Comprehensive prospective data is essential to optimize resource allocation and identify both modifiable and non-modifiable risk factors at the patient, surgical, and hospital levels. As such, we conducted PakSurg 1, a prospective, multicentre study spanning various surgical subspecialties across Pakistan. By collecting comprehensive data from diverse provinces and cities, we aimed to determine the incidence of SSIs in Pakistan across different surgical subspecialties, assess the existing local preoperative, intraoperative, and postoperative practices for prevention of SSI, and evaluate the risk factors for the development of SSIs.

## Methods

### Study design

PakSurg 1 was designed as a trainee-led, national, multicentre, prospective cohort study where patient recruitment was conducted from September 20, 2022, to September 30, 2023, with a follow-up period extending to October 30, 2023. This investigation was carried out across 28 secondary and tertiary healthcare facilities within Pakistan, including public and private institutions. Coordination of investigators at each participating centre was managed by a national steering committee, which ensured rigorous adherence to standardized protocols for patient recruitment and data collection. A publicly available protocol detailing the methodology has been previously published by our team.^18^

### Participants

Adult patients aged 18 years and older scheduled for elective surgeries in designated subspecialties, including breast, cardiac, colorectal, cranial, general, obstetrics and gynecology, orthopaedic, spine, and vascular surgery, were eligible for inclusion. Only procedures listed in the protocol's predefined appendix were considered. Patients were excluded if they had preoperative infections, experienced intraoperative mortality, or lacked 30-day postoperative follow-up data. Emergency procedures were excluded to reduce clinical heterogeneity and allow evaluation of modifiable perioperative factors under standardized conditions. Emergency surgery often involves higher contamination levels, limited preoperative optimization, urgent antibiotic administration, and increased loss to follow-up, which could confound assessment of system-level preventive practices.^19^

### Procedures

Data were captured using digital data entry forms designed using the Research Electronic Data Capture (REDCap) system, access to which was provided by AKU, Karachi.^20^

The data collection tools were self-designed by the PakSurg 1 steering committee after intensive literature review, and have been included in Supplementary Appendix 1.^18^ The questionnaire was developed in English, given that English is the primary language of documentation across hospitals in Pakistan. To ensure validity, the questionnaire was reviewed for face validity by multiple surgeons at AKU, confirming that it appropriately measured the intended constructs. We also conducted a pilot test using 10 surgical patients from AKU to rectify errors and troubleshoot potential pitfalls in data collection. Given that no major errors were found, the questionnaire did not undergo substantive changes after the pilot test. All operational definitions have been specified previously.^18^

Potential institutional collaborators were identified through purposive and snowball sampling. Information about PakSurg 1 was disseminated via official institutional email addresses of all medical colleges registered with the Pakistan Medical and Dental Council (PMDC), as well as through social media platforms such as WhatsApp, Facebook, and Instagram.

At each institution, multiple mini-teams, each consisting of three collaborators, were assigned sequentially, with each team collecting data on all consecutive, eligible patients over a 1-month period.^18^

Data collection was prospective, beginning in the preoperative phase and continuing through 30 days post-surgery for each patient. Data was manually gathered using the anonymized, digitized data collection tool on prespecified variables comprising patient demographics, operative details, and postoperative outcomes, including SSI incidence classified according to the 2023 Centres for Disease Control and Prevention (CDC) definitions, by reviewing all available patient documentation and medical records.^21^ Body mass index (BMI) measurements were categorized according to the South Asian criteria.^22^, ^23^, ^24^ Surgical wounds were classified according to the CDC surgical wound classification system i.e. clean (Class I), clean-contaminated (Class II), contaminated (Class III), or dirty (Class IV).^25^

Follow-up was conducted in accordance with the prespecified protocol, involving inpatient observation, discharge evaluation, and telephonic interviews on postoperative days 3, 15, and 30.^18^ Hospital- and department-level data were collected upon the registration of data collectors from participating institutions.

Site-specific collaborators were granted access to REDCap via data access groups, allowing them to submit and view data only from their respective teams. Each patient record was automatically assigned a REDCap ID, and collaborators maintained a local encrypted Excel sheet to link REDCap IDs to patient identifiers. Quality checks were embedded within the REDCap form to minimize entry errors. Data submission was fully anonymized, with no patient or hospital identifiers transmitted.

The initial dataset of 2165 surgeries was cleaned to ensure strict adherence to the prespecified eligibility criteria, yielding 1762 surgeries (Supplementary Appendix 2, Supplementary Fig. S1). Multiple records (n = 51) were found to have missing data. Little's test for missing completely at random (MCAR) rejected the null hypothesis (χ^2^ = 9,685,270.7, df = 181, p < 0.001), indicating that missingness was unlikely to be MCAR. However, given that missing data constituted fewer than 5% of eligible records, prospective data collection using standardized forms, and absence of clear patterns suggesting outcome-dependent missingness, we opted to perform a complete case analysis (CCA) rather than impute the missing data.^26^

### Ethics

Ethics approval was obtained from the institutional review board at the Aga Khan University (AKU) in Karachi, (ERC#2022-7771-22827) and from the Pakistan National Institutes of Health National Bioethics Committee (NBC-862/22/533); all participating institutions that were recruited thereafter obtained ethical approval from their own institutional review boards. All patients provided written informed consent before enrolment.^18^

### Statistical analysis

All statistical analyses were performed using International Business Machines (IBM) Statistical Package for Social Sciences (SPSS) version 26. Categorical variables were summarized as frequencies and percentages. Continuous variables were checked for normality visually using histograms and quantile–quantile (Q–Q) plots, and the Kolmogorov–Smirnov test was used as an adjunct statistical test. Parametrically distributed continuous variables were summarized as means and standard deviations, while non-parametrically distributed continuous variables were summarized as medians and interquartile ranges (IQRs).

Differences between patients that developed SSIs and those that did not were evaluated using the chi-square (χ^2^) test or Fisher's exact test for categorical variables. Parametrically and non-parametrically distributed variables were compared between the two groups using student's t-test for independent samples and the Mann–Whitney U test, respectively.

Multivariable binary logistic regression models were employed to analyse the association between development of SSI within 30 days of surgery and various preoperative, intraoperative, and postoperative covariates. Variables were selected into the multivariable model using a threshold of p < 0.05 on univariate binary logistic regression analysis. All variables meeting this criterion were assessed for multicollinearity before final inclusion in the multivariable model, whereby a variance inflation factor (VIF) value of >5 was used as the threshold to establish multicollinearity and subsequent exclusion from the multivariable model. Results were presented as odds ratios (ORs) with 95% confidence intervals (CIs). The threshold for statistical significance for all analyses was p < 0.05.

As a sensitivity analysis to assess robustness of variable selection and reduce the risk of model-dependent associations, we fitted penalized logistic regression using Elastic Net (logit link) with SSI as the binary outcome. This was implemented in Python 3.13 using the scikit-learn library. Elastic Net combines Least Absolute Shrinkage and Selection Operator (LASSO) and ridge penalties, enabling simultaneous coefficient shrinkage and variable selection while maintaining stability in the presence of correlated predictors, which is common in clinical datasets.^27^ Hyperparameters were selected using stratified 10-fold cross-validation to preserve the proportion of SSI events in each split. In each iteration, the model was trained on nine folds and evaluated on the held-out fold, and performance was averaged across folds to minimize overfitting. The penalty strength (C) and Elastic Net mixing parameter (α; l1_ratio) were tuned to maximize the area under the receiver operating characteristic curve (AUC). Coefficient magnitudes from the final penalized model were used as a measure of relative variable importance, with larger absolute standardized coefficients indicating greater contribution to prediction. Because length of stay (LOS) may be influenced by postoperative complications including SSI (and may therefore act as a mediator or consequence rather than a purely preoperative predictor), we also repeated Elastic Net excluding LOS.

The National Nosocomial Infections Surveillance (NNIS) risk index was calculated as an additional metric to assess the risk of SSI as per our prespecified protocol.^28^ The NNIS risk index assigns a score of 1 to each of the following: contaminated/dirty wound, operative duration >75th percentile of the procedure, and an ASA status >3. However, given that some previous studies have questioned the appropriateness of the NNIS as a composite measure in certain surgical subspecialties,^29^^,^^30^ and that the difference in NNIS risk index score between the SSI and non-SSI groups was not found to be statistically significant, the composite measure was not included in further analyses.

### Role of funding source

This study received monetary funding from the Student & Trainee Initiated Research Program (MBBS Class of ’88 Awards, Aga Khan University, Karachi, Pakistan). This funding was used for obtaining necessary national approvals from the National Bioethics Committee, and dissemination of information and resources related to PakSurg 1. Funding played no role in data collection, analysis, or interpretation; trial design; patient recruitment; or any aspect pertinent to the study.

## Results

### Participating healthcare facilities

Data from 28 healthcare facilities were included. Most centres were located in Punjab (50.0%; n = 14), followed by Sindh (21.4%; n = 6) and Khyber Pakhtunkhwa (17.9%; n = 5). The majority were tertiary (96.4%; n = 27), teaching (96.4%; n = 27), and public institutions (75.0%; n = 21). The median number of hospital beds was 705 (IQR: 500–1475). At baseline, 53.6% (n = 15) of hospitals conducted routine 30-day follow-up, all via outpatient clinics. Obstetrics and gynaecology (82.1%; n = 23) and general surgery (78.6%; n = 22) were the most commonly represented subspecialties, while vascular surgery was least represented (10.7%; n = 3). Additional institutional data, including number of cases contributed, average number of eligible surgeries per month, and number of surgical trainees and faculty per specialty are reported for each institution in Supplementary Appendix 2, Supplementary Tables S1–S5.

### Baseline patient characteristics

A total of 1711 patients were included (Table 1). The mean age was 44.4 ± 15.5 years, and 67.3% (n = 1151) were female. Most patients were obese (55.8%; n = 954) and had an ASA class <3 (85.0%; n = 1455). The majority were non-diabetic (80.9%; n = 1385), never smokers (88.4%; n = 1512), and not using corticosteroids preoperatively (95.4%; n = 1633). Preoperative immunosuppressant use (6.1% vs. 4.4%; p = 0.028) and chemotherapy (6.7% vs. 2.2%; p = 0.002) were more common among patients who developed SSIs.

**Table 1:** Baseline patient characteristics.

Variable	Categories	Total (n = 1711)	No SSI (n = 1548)	SSI (n = 163)	p-value
Age (Years)	–	44.43 ± 15.52	44.37 ± 15.62	45.05 ± 14.59	0.575
Age (Years)—categorized	18–39	724 (42.3)	664 (42.9)	60 (36.8)	0.099
	40–59	647 (37.8)	572 (37.0)	75 (46.0)	
	60–80	320 (18.7)	295 (19.1)	25 (15.3)	
	80+	20 (1.2)	17 (1.1)	3 (1.8)	
Biological sex	Male	560 (32.7)	513 (33.1)	47 (28.8)	0.265
	Female	1151 (67.3)	1035 (66.9)	116 (71.2)	
Height (cm)	–	162.81 ± 9.18	162.69 ± 9.22	163.93 ± 8.69	0.102
Weight (kg)	–	69.61 ± 13.34	69.58 ± 13.33	69.88 ± 13.43	0.783
Body mass index (kg/m^2^)	–	26.37 ± 5.29	26.40 ± 5.28	26.12 ± 5.43	0.529
Body mass index—categorized	Underweight	57 (3.3)	52 (3.4)	5 (3.1)	0.129
	Normal	382 (22.3)	345 (22.3)	37 (22.7)	
	Overweight	318 (18.6)	277 (17.9)	41 (25.2)	
	Obese	954 (55.8)	874 (56.5)	80 (49.1)	
American society of anesthesiology classification	Class I	898 (52.5)	816 (52.7)	82 (50.3)	0.261
	Class II	557 (32.6)	497 (32.1)	60 (36.8)	
	Class III	161 (9.4)	144 (9.3)	17 (10.4)	
	Class IV	94 (5.5)	90 (5.8)	4 (2.5)	
	Class V	1 (0.1)	1 (0.1)	0 (0.0)	
HIV seropositivity	Positive	0 (0.0)	0 (0.0)	0 (0.0)	0.222
	Negative	1620 (94.7)	1469 (94.9)	151 (92.6)	
	Unknown	91 (5.3)	79 (5.1)	12 (7.4)	
Type 2 diabetes mellitus	Yes (Lifestyle-Controlled)	56 (3.3)	50 (3.2)	6 (3.7)	0.345
	Yes (Non-Insulin Dependent Medication-Controlled)	174 (10.2)	152 (9.8)	22 (13.5)	
	Yes (Insulin-Dependent)	96 (5.6)	90 (5.8)	6 (3.7)	
	No	1385 (80.9)	1256 (81.1)	129 (79.1)	
Preoperative corticosteroid use	Yes	78 (4.6)	68 (4.4)	10 (6.1)	0.310
	No	1633 (95.4)	1480 (95.6)	153 (93.9)	
Preoperative immunosuppressant use	Yes	19 (1.1)	14 (0.9)	5 (3.1)	**0.028**
	No	1692 (98.9)	1534 (99.1)	158 (96.9)	
Preoperative chemotherapy	Yes	45 (2.6)	34 (2.2)	11 (6.7)	**0.002**
	No	1666 (97.4)	1514 (97.8)	152 (93.3)	
Smoking status	Never Smoked	1512 (88.4)	1371 (88.6)	141 (86.5)	0.100
	Ex-Smoker	108 (6.3)	98 (6.4)	10 (6.2)	
	Current Smoker	91 (5.3)	79 (5.1)	12 (7.4)	
Tuberculosis	Recent Diagnosis (≤9 Months)	5 (0.3)	5 (0.3)	0 (0.0)	0.625
	Past Diagnosis (>9 Months)	31 (1.8)	27 (1.7)	4 (2.5)	
	Never Diagnosed	1675 (97.9)	1516 (97.9)	159 (97.5)	
Source of patient identification for study inclusion	Operating Theatre Logbook Review	168 (9.8)	146 (9.4)	22 (13.5)	0.059
	Scheduled Surgery Lists/Diaries	771 (45.1)	703 (45.4)	68 (41.7)	
	Clinical Handover Records	7 (0.4)	7 (0.5)	0 (0.0)	
	Ward Roster/Admission List Review	447 (26.1)	414 (26.7)	33 (20.2)	
	Multiple Sources	318 (18.6)	278 (18.0)	40 (24.5)	

**Note:** All categorical variables have been represented as frequencies and percentages of the column total, while continuous variables have been represented as means and standard deviations or medians and interquartile ranges where appropriate. p-values that are less than 0.05 have been made **bold**.

Statistical comparisons for categorical variables have been made using the chi-square (χ^2^) test or Fisher's exact test. Statistical comparisons between groups have been made using Student's t-test for independent samples for parametrically distributed continuous variables, and Mann–Whitney U test for non-parametrically distributed continuous variables.

Abbreviation: SSI: Surgical Site Infection.

### Preoperative surgical planning and patient preparation

Most patients underwent general surgical (44.0%; n = 752) or obstetrics and gynaecological (27.6%; n = 473) procedures. A detailed count of all surgical procedures included in the final analysis is provided in Supplementary Appendix 2, Supplementary Table S6. The majority of participants received surgery for a benign indication (69.5%; n = 1190), while the development of SSI was associated with malignant (SSI: 12.3% vs. non-SSI: 5.2%; p = 0.002) and operations performed for traumatic injuries (SSI: 8.6% vs. non-SSI: 6.8%; p = 0.002).

Preoperative hair removal was performed in most patients, most commonly using a razor (45.6%; n = 780), followed by clippers (17.9%; n = 306). Pre-incision skin preparation was most frequently performed using povidone-iodine (68.1%; n = 1165). Use of combined povidone-iodine and chlorhexidine was more common in patients without SSIs (15.8% vs. 4.9%; p < 0.001). Similarly, complete skin drying (94.5% vs. 89.0%; p = 0.005) and surgical site marking (74.4% vs. 65.6%; p = 0.016) were more frequent among patients without SSIs. Patients who received preoperative antibiotics (62.0% vs. 52.1%; p = 0.017) and antibiotic prophylaxis at incision (53.4% vs. 44.4%; p = 0.028) were more likely to develop SSIs (Table 2).

**Table 2:** Preoperative surgical planning and patient preparation.

Variable	Categories	Total (n = 1711)	No SSI (n = 1548)	SSI (n = 163)	p-value
Surgical indication	Benign	1190 (69.5)	1086 (70.2)	104 (63.8)	**0.002**
	Malignant	101 (5.9)	81 (5.2)	20 (12.3)	
	Trauma	120 (7.0)	106 (6.8)	14 (8.6)	
	Obstetric	300 (17.5)	275 (17.8)	25 (15.3)	
Surgical intent (For Malignant Indications) *N = 101*	Curative	88 (87.1)	71 (87.7)	17 (85.0)	0.932
	Palliative	8 (7.9)	6 (7.4)	2 (10.0)	
	Surgical Diagnosis	5 (5.0)	4 (4.9)	1 (5.0)	
Preoperative hair removal	Yes (Razor Used)	780 (45.6)	703 (45.4)	77 (47.2)	0.670
	Yes (Clippers Used)	306 (17.9)	281 (18.2)	25 (15.3)	
	No	625 (36.5)	564 (36.4)	61 (37.4)	
Location of hair removal *N = 1086*	Home	275 (25.3)	243 (24.7)	32 (31.4)	0.064
	Ward	389 (35.8)	363 (36.9)	26 (25.5)	
	Operating Room	422 (38.9)	378 (38.4)	44 (43.1)	
Pre-incision skin preparation	Chlorhexidine	293 (17.1)	255 (16.5)	38 (23.3)	**<0.001**
	Povidone-Iodine	1165 (68.1)	1048 (67.7)	117 (71.8)	
	Both	253 (14.8)	245 (15.8)	8 (4.9)	
Skin fully dried before incision	Yes	1608 (94.0)	1463 (94.5)	145 (89.0)	**0.005**
	No	103 (6.0)	85 (5.5)	18 (11.0)	
Surgical site marked	Yes	1258 (73.6)	1151 (74.4)	107 (65.6)	**0.016**
	No	452 (26.4)	396 (25.6)	56 (34.4)	
Preoperative antibiotics used (last dose within 24 hours of surgery)	Yes	908 (53.1)	807 (52.1)	101 (62.0)	**0.017**
	No	803 (46.9)	741 (47.9)	62 (38.0)	
Duration on antibiotics before surgery (Days) *N = 908*	–	1 [1–2]	1 [1–2]	1 [1–3]	0.075
Antibiotic prophylaxis at point of incision	Yes	774 (45.2)	687 (44.4)	87 (53.4)	**0.028**
	No	937 (54.8)	861 (55.6)	76 (46.6)	

**Note:** All categorical variables have been represented as frequencies and percentages of the column total, while continuous variables have been represented as means and standard deviations or medians and interquartile ranges where appropriate. p-values that are less than 0.05 have been made **bold**.

Statistical comparisons for categorical variables have been made using the chi-square (χ^2^) test or Fisher's exact test. Statistical comparisons between groups have been made using Student's t-test for independent samples for parametrically distributed continuous variables, and Mann–Whitney U test for non-parametrically distributed continuous variables.

Abbreviation: SSI: Surgical Site Infection.

### Operative characteristics and postoperative course

**Fig. 1:**
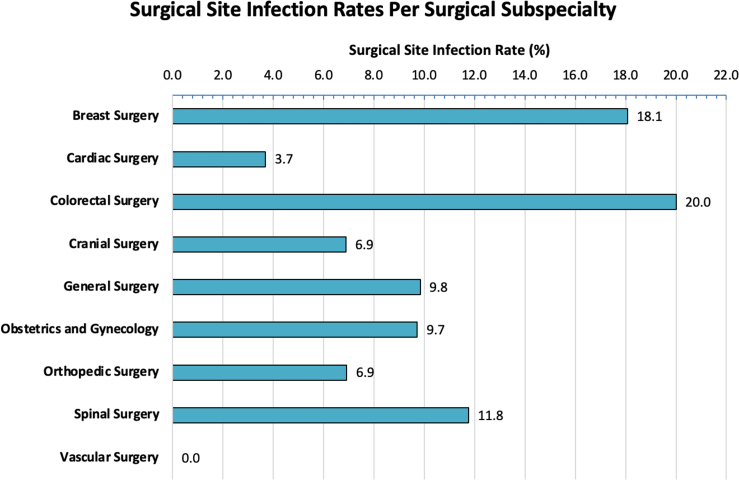
Surgical site infection rates per surgical subspecialty.

Colorectal surgery had the highest proportion of patients who developed SSIs (20.0%; n = 3), followed by breast surgery (18.1%; n = 15) and spinal surgery (11.1%; n = 8). Conversely, the lowest proportion of patients who developed SSIs were of vascular surgery (0.0%; n = 0), and cardiac surgery (3.7%; n = 4). These results are diagrammatically represented in Fig. 1.

In addition, patients who developed SSIs were less likely to have undergone same-day or ambulatory surgery (16.0% vs. 24.5%; p = 0.014) and to have had surgical timeouts performed (84.0% vs. 92.6%; p < 0.001). Open procedures were more common (77.9% vs. 64.2%; p < 0.001), while laparoscopic procedures were less frequent (17.2% vs. 29.2%; p < 0.001). Clean wounds were also less common in the SSI group (59.5% vs. 65.1%; p < 0.001).

ICU admission was required in 8.2% (n = 141) of patients, with a median length of stay of 45 h [IQR: 15–48]. Patients who developed SSIs had a longer postoperative hospital stay (median: 5 vs. 4 days; p < 0.001) and were more likely to receive extended antibiotic prophylaxis (50.3% vs. 39.0%; p = 0.005). Thirty-day mortality occurred in 1.0% (n = 17) of patients, and unplanned reintervention in 0.8% (n = 14). Operative characteristics and postoperative outcomes are summarized in Table 3.

**Table 3:** Operative characteristics and postoperative outcomes.

Variable	Categories	Total (n = 1711)	No SSI (n = 1548)	SSI (n = 163)	p-value
Surgical subspecialty	Breast Surgery	83 (4.9)	68 (4.4)	15 (9.2)	**0.024**
	Cardiac Surgery	108 (6.3)	104 (6.7)	4 (2.5)	
	Colorectal Surgery	15 (0.9)	12 (0.8)	3 (1.8)	
	Cranial Surgery	58 (3.4)	54 (3.5)	4 (2.5)	
	General Surgery	752 (44.0)	678 (43.8)	74 (45.4)	
	Obstetrics and Gynecology	473 (27.6)	427 (27.6)	46 (28.2)	
	Orthopedic Surgery	130 (7.6)	121 (7.8)	9 (5.5)	
	Spinal Surgery	68 (4.0)	60 (3.9)	8 (4.9)	
	Vascular Surgery	24 (1.4)	24 (1.6)	0 (0.0)	
Same-day/ambulatory surgery	Yes	406 (23.7)	380 (24.5)	26 (16.0)	**0.014**
	No	1305 (76.3)	1168 (75.5)	137 (84.0)	
Number of individuals present at start of surgery	–	7 [6–8]	7 [6–8]	6 [6–8]	0.538
Number of individuals present at end of surgery	–	6 [5–8]	7 [5–8]	6 [5–8]	0.526
Time-out before surgery	Yes	1571 (91.8)	1434 (92.6)	137 (84.0)	**<0.001**
	No	140 (8.2)	114 (7.4)	26 (16.0)	
Operative approach	Open Surgery	1121 (65.5)	994 (64.2)	127 (77.9)	**0.004**
	Laparoscopic Surgery	486 (28.4)	458 (29.6)	28 (17.2)	
	Laparoscopic-to-Open Conversion	46 (2.7)	41 (2.6)	5 (3.1)	
	Hybrid Surgery	58 (3.4)	55 (3.6)	3 (1.8)	
Type of anesthesia	Local Anesthesia	19 (1.1)	19 (1.2)	0 (0.0)	0.143
	Nerve Block	4 (0.2)	3 (0.2)	1 (0.6)	
	Spinal	460 (26.9)	421 (27.2)	39 (23.9)	
	Epidural	11 (0.6)	8 (0.5)	3 (1.8)	
	General	1175 (68.7)	1058 (71.8)	117 (71.8)	
	Multiple	42 (2.5)	39 (2.5)	3 (1.8)	
Postoperative epidural analgesia	Yes	110 (6.4)	99 (6.4)	11 (6.7)	0.861
	No	1601 (93.6)	1449 (93.6)	152 (93.3)	
Surgical wound classification	Clean	1105 (64.6)	1008 (65.1)	97 (59.5)	**<0.001**
	Clean-Contaminated	578 (33.8)	522 (33.7)	56 (34.4)	
	Contaminated	26 (1.5)	16 (1.0)	10 (6.1)	
	Dirty/Infected	2 (0.1)	2 (0.1)	0 (0.0)	
Operative time (Minutes)	–	80 [60–120]	80 [60–120]	90 [60–120]	0.295
WHO surgical safety checklist used	Yes	1407 (82.2)	1278 (82.6)	129 (79.1)	0.278
	No	304 (17.8)	270 (17.4)	34 (20.9)	
National nosocomial infections surveillance risk index score	0	1129 (66.0)	1026 (66.3)	103 (63.2)	0.604
	1	508 (29.7)	458 (29.6)	50 (30.7)	
	2	73 (4.3)	63 (4.1)	10 (6.1)	
	3	1 (0.1)	1 (0.1)	0 (0.0)	
ICU admission	Yes	141 (8.2)	127 (8.2)	14 (8.6)	0.865
	No	1570 (91.8)	1421 (91.8)	149 (91.4)	
ICU length of stay (Hours) *N = 141*	–	45 [15–48]	46 [18–48]	24 [10–72]	0.656
Hospital length of stay (Days)	–	4 [3–6]	4 [3–6]	5 [3–10]	**<0.001**
Extended postoperative antibiotic prophylaxis	Yes	686 (40.1)	604 (39.0)	82 (50.3)	**0.005**
	No	1025 (59.9)	944 (61.0)	81 (49.7)	
Duration on antibiotics after surgery (Days) *N = 686*	–	5 [4–7]	5 [4–7]	5 [3–7]	0.097
Nosocomial infection	Yes	25 (1.5)	18 (1.2)	7 (4.3)	**0.007**
	No	1686 (98.5)	1530 (98.8)	156 (95.7)	
Types of nosocomial infection *N = 25*	UTI	15 (60.0)	9 (50.0)	6 (85.7)	0.529
	Pneumonia	5 (20.0)	4 (22.2)	1 (14.3)	
	CLABSI	1 (4.0)	1 (5.6)	0 (0.0)	
	Peripheral Line Infection	1 (4.0)	1 (5.6)	0 (0.0)	
	Others	3 (12.0)	3 (16.7)	0 (0.0)	
30-Day mortality	Yes	17 (1.0)	14 (0.9)	3 (1.8)	0.216
	No	1694 (99.0)	1534 (99.1)	160 (98.2)	
30-Day unplanned reintervention	Yes	14 (0.8)	9 (0.6)	5 (3.1)	**0.007**
	No	1697 (99.2)	1539 (99.4)	158 (96.9)	
Type of unplanned reintervention *N = 14*	Surgical	8 (57.1)	3 (33.3)	5 (100.0)	**0.022**
	Endoscopic	4 (28.6)	4 (44.4)	0 (0.0)	
	Radiological	2 (14.3)	2 (22.2)	0 (0.0)	

**Note:** All categorical variables have been represented as frequencies and percentages of the column total, while continuous variables have been represented as means and standard deviations or medians and interquartile ranges where appropriate. p-values that are less than 0.05 have been made **bold**.

Statistical comparisons for categorical variables have been made using the chi-square (χ^2^) test or Fisher's exact test. Statistical comparisons between groups have been made using Student's t-test for independent samples for parametrically distributed continuous variables, and Mann–Whitney U test for non-parametrically distributed continuous variables.

Abbreviations: SSI: Surgical Site Infection; WHO: World Health Organization; ICU: Intensive Care Unit; UTI: Urinary Tract Infection; CLABSI: Central Line Associated Bloodstream Infection.

### Surgical site infection characteristics

The cumulative incidence of SSIs was 9.5% (n = 163) (Table 4). Most SSIs were diagnosed during the index hospitalization (24.5%; n = 40), and were predominantly superficial incisional (73.6%; n = 120). Management was primarily non-surgical with antibiotics (66.9%; n = 109). A wound swab was obtained in 17.1% (n = 27) of cases; among these, gram-negative bacilli (25.9%; n = 7) and *Staphylococcus aureus* (18.5%; n = 5) were the most commonly identified pathogens.

**Table 4:** Surgical site infection characteristics (N = 163).

Variable	Categories	Number of surgeries
Time of diagnosis	During Index Hospitalization	40 (24.5)
	At Readmission	10 (6.1)
	At Post-Discharge Outpatient Follow-Up	49 (30.1)
	At Post-Discharge Telephonic Follow-Up	64 (39.3)
Type of surgical site infection	Superficial Incisional	120 (73.6)
	Deep Incisional	40 (24.5)
	Organ/Space	3 (1.2)
Organ/space site *N = 3*	Bone	1 (33.3)
	Gastrointestinal	2 (66.7)
Treatment for surgical site infection	Operative Drainage/Debridement	18 (10.0)
	Antibiotic Therapy	109 (66.9)
	Both	36 (22.1)
30-Day readmission due to surgical site infection	Yes	30 (18.4)
	No	133 (81.6)
Wound swab sent	Yes	27 (17.1)
	No	131 (82.9)
Culture result *N = 27*	Gram Negative Bacilli	7 (25.9)
	Staphylococcus Aureus	5 (18.5)
	Anaerobes	2 (8.3)
	Pseudomonas Aeruginosa	2 (8.3)
	Candida Albicans	1 (4.2)
	Others	8 (29.6)
	Multiple	2 (8.3)
Bacterial sensitivity *N = 27*	Sensitive to At Least One Drug	12 (44.4)
	Pan-Resistant	1 (3.7)
	Sensitivity Not Tested	14 (51.9)

### Predictors of surgical site infection incidence

Independent predictors of SSIs on multivariable binary logistic regression included surgical indication of trauma (OR: 3.047 [1.205–7.708]; p = 0.019), a contaminated or dirty surgical wound (OR: 3.245 [1.298–8.113]; p = 0.012), and an increased hospital LOS (OR: 1.068 [1.036–1.101]; p < 0.001).

Factors that were independently associated with decreased incidence of SSIs included ambulatory surgery (OR: 0.598 [0.375–0.953]; p = 0.030), use of both chlorhexidine and povidone-iodine for preoperative skin preparation compared with either individual agent (OR: 0.304 [0.131–0.704]; p = 0.005), implementing a surgical time-out (OR: 0.446 [0.268–0.743]; p = 0.002), and laparoscopic surgery compared to open surgery (OR: 0.556 [0.343–0.900]; p = 0.017). Additionally, our sample showed that cardiac (OR: 0.223 [0.074–0.672]; p = 0.008), cranio-spinal (OR: 0.292 [0.126–0.676]; p = 0.004), and orthopaedic (OR: 0.216 [0.071–0.655]; p = 0.007) surgical procedures were less likely to develop an SSI compared to general surgical procedures. These results are presented in Table 5.

**Table 5:** Binary logistic regression analysis for factors associated with incidence of surgical site infections.

Variable	Categories	Univariable odds ratio [95% CI]	p-value	Multivariate odds ratio [95% CI]	p-value
Age (Years)	–	1.003 [0.993–1.013]	0.595		
Biological sex	Female	Reference	–		
	Male	0.817 [0.573–1.166]	0.266		
Body mass index^c^	Normal Weight	Reference	–		
	Underweight	0.897 [0.337–2.385]	0.827		
	Overweight	1.380 [0.861–2.212]	0.181		
	Obese	0.853 [0.567–1.285]	0.448		
American society of anesthesiology classification	ASA Class 1 or 2	Reference	–		
	ASA Class ≥3	0.826 [0.512–1.334]	0.435		
Type 2 diabetes mellitus	No	Reference	–		
	Yes	1.134 [0.761–1.689]	0.537		
Preoperative corticosteroid use	No	Reference	–		
	Yes	1.423 [0.718–2.820]	0.313		
Preoperative immunosuppressant use	No	Reference	–	Reference	–
	Yes	3.467 [1.233–9.753]	**0.018**	1.901 [0.571–6.330]	0.296
Preoperative chemotherapy	No	Reference	–	Reference	–
	Yes	3.223 [1.600–6.489]	**0.001**	1.736 [0.681–4.428]	0.248
Smoking status	No	Reference	–		
	Yes	1.209 [0.751–1.945]	0.435		
History of tuberculosis	No	Reference	–		
	Yes	1.192 [0.416–3.413]	0.744		
Same-day/ambulatory surgery	No	Reference	–	Reference	–
	Yes	0.583 [0.378–0.901]	**0.015**	0.598 [0.375–0.953]	**0.030**
Surgical indication	Benign Disease	Reference	–	Reference	–
	Malignancy	2.578 [1.519–4.337]	**<0.001**	1.300 [0.634–2.667]	0.474
	Trauma	1.379 [0.763–2.494]	0.288	3.047 [1.205–7.708]	**0.019**
	Obstetrics	0.949 [0.602–1.498]	0.823	0.693 [0.362–1.327]	0.269
Preoperative antibiotics used (last dose within 24 hours of surgery)	No	Reference	–	Reference	–
	Yes	1.496 [1.074–2.084]	**0.017**	1.229 [0.641–2.357]	0.535
Preoperative hair removal	No	Reference	–		
	Yes (With Razor)	1.013 [0.711–1.443]	0.944		
	Yes (With Clipper)	0.823 [0.505–1.339]	0.432		
Pre-incision skin preparation	Chlorhexidine	Reference	–	Reference	–
	Povidone-Iodine	0.749 [0.507–1.107]	0.147	0.687 [0.444–1.061]	0.090
	Both	0.219 [0.100–0.479]	**<0.001**	0.304 [0.131–0.704]	**0.005**
Skin fully dried before incision	No	Reference	–	Reference	–
	Yes	0.468 [0.274–0.800]	**0.006**	0.737 [0.396–1.373]	0.336
Number of individuals present at start of surgery	–	0.999 [0.998–1.001]	0.397		
Number of individuals present at end of surgery	–	0.999 [0.998–1.001]	0.399		
Surgical site marked	No	Reference	–	Reference	–
	Yes	0.657 [0.467–0.926]	**0.016**	0.858 [0.583–1.261]	0.435
Surgical time-out	No	Reference	–	Reference	–
	Yes	0.419 [0.264–0.664]	**<0.001**	0.446 [0.268–0.743]	**0.002**
Operative approach	Open Surgery	Reference	–	Reference	–
	Laparoscopic Surgery	0.478 [0.313–0.731]	**0.001**	0.556 [0.343–0.900]	**0.017**
	Laparoscopic-to-Open Conversion	0.954 [0.370–2.640]	0.923	1.225 [0.439–3.423]	0.698
	Hybrid Surgery	0.427 [0.132–1.385]	0.156	0.312 [0.084–1.153]	0.081
Type of anesthesia	General	Reference	–		
	Multimodal	0.696 [0.212–2.286]	0.696		
	Non-General	0.862 [0.598–1.244]	0.862		
Postoperative epidural analgesia	No	Reference	–		
	Yes	1.059 [0.556–2.019]	0.861		
Surgical wound classification	Clean	Reference	–	Reference	–
	Clean-Contaminated	1.115 [0.789–1.575]	0.537	0.986 [0.665–1.463]	0.945
	Contaminated/Dirty	5.773 [2.592–12.856]	**<0.001**	3.245 [1.298–8.113]	**0.012**
Antibiotic prophylaxis at point of incision	No	Reference	–	Reference	–
	Yes	1.435 [1.038–1.983]	**0.029**	1.110 [0.583–2.111]	0.751
Extended postoperative antibiotic prophylaxis^a^	No	Reference	–	–	–
	Yes	1.582 [1.145–2.187]	**0.005**	–	–
Operative duration (Minutes)	–	1.000 [0.998–1.002]	0.715		
WHO surgical safety checklist used	No	Reference	–		
	Yes	0.802 [0.537–1.196]	0.278		
ICU admission	No	Reference	–		
	Yes	1.051 [0.590–1.872]	0.865		
Hospital length of stay (days)	–	1.055 [1.031–1.079]	**<0.001**	1.068 [1.036–1.101]	**<0.001**
Surgical specialty	General Surgery^b^	Reference	–	Reference	–
	Cardiac Surgery	0.317 [0.114–0.880]	**0.028**	0.223 [0.074–0.672]	**0.008**
	Cranio-Spinal Surgery	0.867 [0.461–1.633]	0.659	0.292 [0.126–0.676]	**0.004**
	Obstetrics and Gynecology	0.888 [0.611–1.289]	0.531	0.912 [0.526–1.581]	0.743
	Orthopedic Surgery	0.613 [0.301–1.248]	0.177	0.216 [0.071–0.655]	**0.007**

Variables were included in the multivariable regression model based on the univariable binary logistic regression showing a statistically significant result (p < 0.005). Bold values represent statistically significant p-values (<0.05).

Abbreviations: CI: Confidence Interval; WHO: World Health Organization.

a Not included in the multivariable regression model because of collinearity (Variance Inflation Factor >5.0) with preoperative antibiotic use and point-of-incision antibiotic prophylaxis.

b Due to a small sample size, vascular surgery and breast surgery were combined with general surgery for the binary logistic regression analysis.

c We performed an additional univariable logistic regression using BMI as a continuous predictor. BMI was not associated with SSI (OR per kg/m^2^ increase 0.99 [95% CI: 0.96–1.02]; p = 0.53), and was thus not carried forward into the multivariable model.

In the Elastic Net sensitivity analysis including LOS, cross-validated discrimination was AUC 0.677 ± 0.062. Because LOS may reflect postoperative course and could act as a mediator or consequence of SSI, we repeated the analysis excluding LOS. Discrimination decreased modestly but remained robust with an AUC of 0.650 ± 0.057, and the principal perioperative predictors remained prominent. These findings support the stability of the primary per-protocol analysis. Coefficients from the penalized model have been tabulated in Supplementary Appendix 2, Supplementary Table S7.

A graphical summary of the study design and key findings is shown in Supplementary Appendix 2, Supplementary Fig. S2.

## Discussion

PakSurg 1 is a large, multicentre, prospective cohort study examining the epidemiology and risk factors associated with SSIs among elective surgical patients in Pakistan. Although several perioperative practices associated with SSI risk are well described in high-income settings, their adoption and effectiveness in LMIC contexts remain variable. This study provides context-specific evidence from routine clinical practice across Pakistan, identifying feasible targets for quality improvement where standardized infection-prevention programs are not uniformly implemented. Our study found a significantly elevated SSI incidence rate of 9.5%, substantially exceeding international benchmarks, which typically range between 0.5% and 3.0%.^31^ We found that the use of multiple antiseptic agents, ambulatory surgery, laparoscopic surgery, and the implementation of a surgical time-out were independently associated with reduced odds of developing an SSI. In contrast, surgical indication of trauma, contaminated surgical wound, and an increased hospital LOS were independently associated with increased odds of developing an SSI. To our knowledge, this is the largest prospective study of SSI prevalence and predictors in Pakistan, offering key evidence to inform policy and improve surgical outcomes.

The observed cumulative SSI rate of 9.5% in our study markedly surpasses the global pooled incidence of 2.5% reported by Dechasa et al.'s 2023 meta-analysis.^32^ However, this rate aligns with findings from studies conducted in low- and middle-income countries (LMICs) comparable to Pakistan, where pooled SSI rates range from 5% to 10%.^33^^,^^34^ Such consistently elevated rates in LMICs point to underlying systemic barriers: overcrowded hospitals, limited access to sterilization equipment, variable implementation of infection control practices, and frequent shortages of trained staff or basic supplies.^35^ In many instances, antibiotic stewardship programs and surveillance mechanisms are also less established, allowing for higher rates of both infection and antimicrobial resistance.^36^^,^^37^ This highlights the significant challenge SSIs pose to healthcare quality and the advancement of surgical care in resource-constrained settings.^38^^,^^39^ Given Pakistan's limited insurance coverage and substantial out-of-pocket healthcare expenses, SSIs impose considerable financial burdens. SSIs not only increase the direct costs of care (additional procedures, medications, laboratory testing, and nursing care) but also drive up indirect costs such as lost income due to extended recovery periods, caretaking responsibilities, and in some cases, permanent disability.^3^ As such, there is a pressing need to incorporate focused SSI reduction strategies into national surgical guidelines. It is worth noting, however, that our aggregate estimated rate of 9.5% reflects a heterogeneous mix of procedures with widely varying baseline infection risks, with higher-risk operations and contaminated wounds likely contributing disproportionately to the burden.

We found that the use of multiple antiseptic agents prior to surgery was significantly associated with a lower SSI rate, supporting evidence that a multimodal approach, such as combining chlorhexidine and povidone-iodine, offers broader antimicrobial protection and more effective skin decontamination than single-agent protocols.^40^ This is in contrast to the National Guidelines for Infection Prevention and Control (NGIPC) Pakistan from 2020, which encourage the use of chlorhexidine-based solutions, but not a multiagent approach.^41^ Similarly, patients undergoing ambulatory surgery experienced markedly fewer SSIs, likely owing to the less complex nature of these procedures and reduced exposure to hospital-acquired pathogens, highlighting the benefits of minimizing hospital stays. Laparoscopic surgery was also linked to reduced SSI risk, as its minimally invasive technique limits tissue disruption and shortens recovery times, thereby decreasing opportunities for infection. However, this association may be partially attributable to selection bias, as patients at lower baseline risk are more likely to undergo laparoscopic procedures. Along this vein, an extended hospital LOS emerged as an independent risk factor for SSIs. Our study found that every additional day spent at the hospital was associated with 6.8% higher odds of developing an SSI. This underlines the importance of strategies such as enhanced recovery protocols to shorten inpatient duration and mitigate infection risks.

An interesting finding of our study was the protective effect of preoperative surgical time-outs in reducing the risk of surgical site infections. Notably, surgical time-outs were implemented in 91.8% of procedures in our sample, reflecting widespread adoption of this practice. Although the evidence directly linking surgical time-outs to SSI rates remains limited, multiple studies have highlighted that this structured pause facilitates timely administration of prophylactic antibiotics, an established measure for preventing SSIs.^31^^,^^42^ The time-out process also reinforces team communication, clarifies roles, and ensures that essential infection control steps are not overlooked. Given the relative ease and low cost of implementation, particularly in resource-limited settings like Pakistan, institutionalizing surgical time-outs represents a practical, high-impact strategy to enhance surgical safety and reduce the burden of SSIs. This also aligns with the recommendations made in the NGIPC Pakistan from 2020, which encourage the use of prophylactic antibiotics that are to be administered within 120 min before incision.^41^

A compelling example of the impact of structured perioperative interventions comes from the “Clean Cut” program in Ethiopia, where the implementation of six key infection prevention standards, such as proper antibiotic timing, skin antisepsis, and use of surgical checklists, led to a statistically significant reduction in SSI rates without major resource investments.^43^ This initiative demonstrates how contextually adapted, process-driven approaches can produce measurable improvements in surgical outcomes in low-resource settings, and further reinforces the value of adopting similar bundled strategies in Pakistan's surgical care.

In addition, surgeries performed for traumatic indications and procedures involving contaminated wounds were both unsurprisingly associated with a significantly higher risk of developing SSIs. Trauma patients frequently present with complex injuries characterized by contaminated wounds, devitalized tissue, foreign material, and increased physiological stress, all of which predispose to postoperative infection and can overwhelm even the most rigorous aseptic techniques. These challenging scenarios underscore the need for heightened vigilance, robust infection control practices, and carefully tailored perioperative strategies, including meticulous wound debridement, judicious antibiotic use, and close postoperative monitoring, to effectively mitigate SSI risk in this patient group.

Overall, PakSurg 1 has identified important risk factors for SSIs and evaluated preventive interventions, emphasizing the need to refine and strengthen existing national surgical and infection prevention guidelines. Beyond quantifying incidence, the study highlights modifiable perioperative practices that are directly amenable to intervention. For frontline clinical teams, adherence to surgical safety processes such as time-outs, optimized skin-preparation strategies, and selection of minimally invasive approaches where feasible represent practical targets. At the institutional level, hospitals can implement standardized infection-prevention protocols, strengthen perioperative training, and expand ambulatory surgical pathways to reduce exposure to nosocomial risk. At the policy level, establishing a national baseline enables monitoring of surgical quality, development of context-appropriate guidelines, and prioritization of resources for infection-prevention programs. This study provides prospective, multicentre epidemiologic data on SSIs in Pakistan, offering essential baseline evidence in a setting without routine national surveillance. Nevertheless, further comprehensive research is required to clarify uncertainties regarding specific preventive measures and to generate evidence-based, context-appropriate recommendations aimed at effective mitigation of SSI rates and, by extension, their substantial medical and economic impacts.

Our study has several limitations. While we were able to estimate the national burden of SSIs, we did not assess the direct financial costs that were associated with these infections. Additionally, PakSurg 1 had a varying number of public and private institutions participating due to recruitment issues, as well as variable amounts of data collected from the various institutions that did participate; this may have led to significant sampling bias. Furthermore, participating hospitals were recruited using purposive and snowball sampling, resulting in overrepresentation of tertiary teaching centres. This limits generalizability to secondary-care and rural facilities, where surgical case mix and infection-prevention capacity may differ. The direction of potential bias is uncertain, as tertiary centres perform more complex procedures but may also have stronger infection-control practices. Because national data on surgical volume by facility level were unavailable, weighting or adjustment for hospital type was not feasible. Therefore, the findings should be interpreted as primarily reflecting outcomes in major surgical centres, which nonetheless provide a substantial proportion of surgical care in Pakistan. Moreover, certain variables that were included in our per-protocol questionnaire which have been shown to be significantly associated with SSI risk in the literature, including preoperative hand-preparation and preoperative bathing, were not reliably recorded in our dataset and as such had to be excluded from the final analysis. In addition, although Little's test indicated that missing data were unlikely to be completely at random, the overall proportion of missing records was low (<5%). Therefore, the potential for bias introduced by CCA is likely limited. Nevertheless, if missingness was related to unmeasured patient or institutional characteristics, some selection bias or attenuation of effect estimates cannot be excluded. Besides this, this study included only elective surgical procedures, and excluding emergency cases limits generalizability of the incidence estimates to the full surgical population. However, focusing on elective procedures enabled evaluation of modifiable perioperative practices under standardized conditions and facilitated reliable prospective follow-up. Lastly, not all eligible surgical cases performed at participating institutions were captured. Patient enrolment depended on local research team capacity, competing clinical responsibilities, and availability of trained data collectors, reflecting the absence of dedicated audit infrastructure. These operational constraints may result in lower case numbers than institutional surgical volumes and could introduce selection bias if enrolled patients differed systematically from non-enrolled patients. However, consecutive recruitment of eligible patients, standardized training, and centralized data management support the internal validity of the dataset. Generalizability to all surgical activity within each institution should therefore be interpreted with caution.

In conclusion, PakSurg 1 provides national baseline estimates of SSI incidence in Pakistan and shows that SSI rates remain a substantial challenge, exceeding international benchmarks. Our study highlights key risk factors, such as increased hospital LOS and contaminated wounds, as well as protective measures like the use of multiple antiseptics and implementing surgical time-outs. Addressing SSIs will require focused infection control practices, updated national guidelines, and ongoing research. Moving forward, future research should focus on developing and evaluating targeted perioperative protocols for high-risk groups, such as trauma and contaminated wound cases, and on optimizing cost-effective prevention strategies that can be widely implemented.

## Contributors

The PakSurg Steering Committee, including the Central Steering Committee leads (UW, SA) and Faculty Leads (AKS, AU, ABA, EB, MM, HI, MS, MSS, NS, RS, SK, SN, TC, SAE), led the conceptualisation, protocol development, ethical approvals, study oversight, data interpretation, and manuscript preparation, with responsibilities distributed across dedicated teams in line with CRediT taxonomy roles. The Writing and Analysis Team (ASF, WA, RSM, HW, IB, DA, AAM, RDU, MA, MAQ) oversaw conceptualisation, protocol writing, ethics approvals, data analysis, and manuscript drafting. The Operations Team (MU, MUM, SSV, FBA, HG, MK) managed REDCap, training modules, data sharing, and agreements. The Outreach Team (IT, DAK, HR, MJ, SR) coordinated with lead collaborators, recruited centres, and supported data collection. The Dissemination Team (HA, MAR, MJAM, MOA) handled project promotion and results dissemination. Institutional Lead Collaborators and Supervising Consultants coordinated local teams, secured approvals, and ensured centre participation, while Institutional Collaborators identified eligible cases, collected data, and submitted it via REDCap, with Institutional Data Validators conducting protocol-specified validation. All members of the steering committee reviewed and approved the final manuscript. Members of the Writing and Analysis Team directly accessed and verified the underlying study data, conducted the formal analyses, and take responsibility for the integrity and accuracy of the data and analyses presented. The decision to submit the manuscript for publication was made collectively by the Steering Committee leads, Faculty Leads, and the Writing and Analysis Team. Data collectors meeting protocol requirements were included in the corporate authorship list under the PakSurg Collaborative (Supplementary Appendix 3), with individual names, affiliations, and roles listed in an appendix and eligibility for subspecialty-specific publications based on their contributions.

## Data sharing statement

The deidentified datasets used and/or analysed during the current study are available from the corresponding author on reasonable request.

## Declaration of interests

The authors declare that there are no conflicts of interest to disclose.

## References

[1] (2019). Global Spending on Health: A World in Transition.

[2] Monegro A.F., Muppidi V., Regunath H. (2026). StatPearls [Internet].

[3] Jenks P.J., Laurent M., McQuarry S., Watkins R. (2014). Clinical and economic burden of surgical site infection (SSI) and predicted financial consequences of elimination of SSI from an English hospital. J Hosp Infect.

[4] Monahan M., Jowett S., Pinkney T. (2020). Surgical site infection and costs in low- and middle-income countries: a systematic review of the economic burden. PLoS One.

[5] Tanner J., Khan D., Aplin C., Ball J., Thomas M., Bankart J. (2009). Post-discharge surveillance to identify colorectal surgical site infection rates and related costs. J Hosp Infect.

[6] Broex E.C.J., van Asselt A.D.I., Bruggeman C.A., van Tiel F.H. (2009). Surgical site infections: how high are the costs?. J Hosp Infect.

[7] O'Donnell O., van Doorslaer E., Rannan-Eliya R.P. (2008). Who pays for health care in Asia?. J Health Econ.

[8] Ebaidalla E.M., Ali M.E.M. (2019). Determinants and impact of household's out-of-pocket healthcare expenditure in Sudan: evidence from urban and rural population∗. Middle East Dev J.

[9] Costabella F., Patel K.B., Adepoju A.V. (2023). Healthcare cost and outcomes associated with surgical site infection and patient outcomes in low- and middle-income countries. Cureus.

[10] Anon (2018). Surgical site infection after gastrointestinal surgery in high-income, middle-income, and low-income countries: a prospective, international, multicentre cohort study. Lancet Infect Dis.

[11] Allegranzi B., Zayed B., Bischoff P. (2016). New WHO recommendations on intraoperative and postoperative measures for surgical site infection prevention: an evidence-based global perspective. Lancet Infect Dis.

[12] Grover S., Xu M., Jhingran A. (2017). Clinical trials in low and middle-income countries - successes and challenges. Gynecol Oncol Rep.

[13] Wang X., Kattan M.W. (2020). Cohort studies: design, analysis, and reporting. Chest.

[14] Sattar F., Sattar Z., Zaman M., Akbar S. (2019). Frequency of post-operative surgical site infections in a tertiary care Hospital in Abbottabad, Pakistan. Cureus.

[15] Ahmed D., Cheema F.H., Ahmed Y.I. (2011). Incidence and predictors of infection in patients undergoing primary isolated coronary artery bypass grafting: a report from a tertiary care hospital in a developing country. J Cardiovasc Surg.

[16] Tariq A., Ali H., Zafar F. (2018). Assesment of predictor variables and clinical consequences associated with surgical site infection in tertiary care setting, Karachi, Pakistan. Pak J Pharm Sci.

[17] Khan M., Khalil J., Rooh-ul-Muqim (2011). Rate and risk factors for surgical site infection at a tertiary care facility in Peshawar, Pakistan. J Ayub Med Coll Abbottabad.

[18] PakSurg Collaborative (2023). PakSurg 1: determining the epidemiology and risk factors of surgical site infections in Pakistan—a multicentre, prospective cohort study. BMJ Open.

[19] Gagen B., Hall C. (2024). Preventing surgical site infections in emergency general surgery: current strategies and recommendations. Curr Surg Rep.

[20] Harris P.A., Taylor R., Minor B.L. (2019). The REDCap consortium: building an international community of software platform partners. J Biomed Inform.

[21] Anon. SSI | PSC | NHSN | CDC. https://www.cdc.gov/nhsn/psc/ssi/index.html.

[22] Palla A.H., Fatimi A.S., Virani S.S., Fatima S.S. (2023). Cardiovascular disease risk stratification in the Pakistani population with and without metabolic syndrome: a single centre cross-sectional study. PLOS Glob Public Health.

[23] Fatima S.S., Fatimi A.S., Abbas M., Farhat S., Mohammed N. (2025). Methylation patterns of diabetes and obesity susceptibility genes in gestational diabetes mellitus: a cross-sectional analysis from Karachi, Pakistan. Metab Syndr Relat Disord.

[24] Fatima S.S., Fatimi A.S., Abbas M., Khan U.I. (2025). Diabetes and obesity susceptibility genes: a cross-sectional analysis of methylation patterns from Karachi, Pakistan. Postgrad Med J.

[25] National Healthcare Safety Network Surgical site infection event (SSI). https://www.cdc.gov/nhsn/pdfs/pscmanual/9pscssicurrent.pdf.

[26] Ross R.K., Breskin A., Westreich D. (2020). When is a complete-case approach to missing data valid? The importance of effect-measure modification. Am J Epidemiol.

[27] Ogutu J.O., Schulz-Streeck T., Piepho H.-P. (2012). Genomic selection using regularized linear regression models: ridge regression, lasso, elastic net and their extensions. BMC Proc.

[28] Anon Protocol for surgical site infection surveillance with a focus on settings with limited resources. https://www.who.int/publications/i/item/protocol-for-surgical-site-infection-surveillance-with-a-focus-on-settings-with-limited-resources.

[29] Gaynes R.P. (2001). Surgical-site infections (SSI) and the NNIS basic SSI risk index, part II: room for improvement. Infect Control Hosp Epidemiol.

[30] Friedman N.D., Bull A.L., Russo P.L., Gurrin L., Richards M. (2007). Performance of the national nosocomial infections surveillance risk index in predicting surgical site infection in Australia. Infect Control Hosp Epidemiol.

[31] Seidelman J.L., Mantyh C.R., Anderson D.J. (2023). Surgical site infection prevention: a review. JAMA.

[32] Mengistu D.A., Alemu A., Abdukadir Abdi A. (2023). Global incidence of surgical site infection among patients: systematic review and meta-analysis. Inquiry.

[33] Biccard B.M., Madiba T.E., Kluyts H.-L. (2018). Perioperative patient outcomes in the African Surgical Outcomes Study: a 7-day prospective observational cohort study. Lancet.

[34] Allegranzi B., Nejad S.B., Combescure C. (2011). Burden of endemic health-care-associated infection in developing countries: systematic review and meta-analysis. Lancet.

[35] Bardossy A.C., Zervos J., Zervos M. (2016). Preventing hospital-acquired infections in low-income and middle-income countries: impact, gaps, and opportunities. Infect Dis Clin North Am.

[36] Pauwels I., Versporten A., Ashiru-Oredope D. (2025). Implementation of hospital antimicrobial stewardship programmes in low- and middle-income countries: a qualitative study from a multi-professional perspective in the Global-PPS network. Antimicrob Resist Infect Control.

[37] Abbas S. (2024). The challenges of implementing infection prevention and antimicrobial stewardship programs in resource-constrained settings. Antimicrob Steward Healthc Epidemiol.

[38] Ng-Kamstra J.S., Arya S., Greenberg S.L.M. (2018). Perioperative mortality rates in low-income and middle-income countries: a systematic review and meta-analysis. BMJ Glob Health.

[39] Danna D.M. (2018). Hospital costs associated with sepsis compared with other medical conditions. Crit Care Nurs Clin North Am.

[40] Davies B.M., Patel H.C. (2016). Does chlorhexidine and povidone-iodine preoperative antisepsis reduce surgical site infection in cranial neurosurgery?. Ann R Coll Surg Engl.

[41] National Institute of Health Pakistan (2020).

[42] Kefale B., Tegegne G.T., Degu A., Molla M., Kefale Y. (2020). Surgical site infections and prophylaxis antibiotic use in the surgical ward of public hospital in Western Ethiopia: a hospital-based retrospective cross-sectional study. Infect Drug Resist.

[43] Forrester J.A., Starr N., Negussie T. (2021). Clean cut (adaptive, multimodal surgical infection prevention programme) for low-resource settings: a prospective quality improvement study. Br J Surg.

