# Supplementary material for: Epidemiology and risk factors of surgical site infections in elective surgeries in Pakistan (2022–2023): a multicentre, prospective cohort study from PakSurg 1
Source: Lancet Reg Health Southeast Asia. 2026 Jun 1;50:100786. doi: 10.1016/j.lansea.2026.100786 (PMC13253196; doi:10.1016/j.lansea.2026.100786)
Supplement: Supplementary Appendix 1 [file mmc1.pdf]

Paksurg Data Collection Form

Please complete the survey below.

Thank you!

Baseline form

Period start date ____________________

Period end date ____________________

Hospital

◯ A O Clinic
◯ Aadil Hospital
◯ Abbas Institute of Medical Sciences
◯ Abbassi Shaheed Hospital
◯ Adamjee Eye Hospital
◯ Afzal Hospital Jhelum
◯ Aga Khan Extended Family Health Center
◯ Aga Khan Extended Family Health Center
◯ Aga Khan Extended Family Health Center
◯ Aga Khan Extended Family Health Centre
◯ Aga Khan Maternity Centre
◯ Aga Khan Medical Center
◯ Aga Khan Medical Center
◯ Aga Khan Medical Center
◯ Aga Khan Medical Center
◯ Aga Khan University Hospital
◯ Akhter Saeed Trust Hospital
◯ Akram Medical Complex
◯ AKU Garden Town
◯ AKU Kardar
◯ AKU Karimabad
◯ Al Hamd Medical Centre
◯ Al Mumtaz Medical Complex
◯ Al Razi Health Care Pvt Ltd Mm Alam Road
◯ Al Syed Hospital Ahata
◯ Al-Noor Diagnostic Center
◯ Allama Iqbal Memorial Teaching Hospital (AIMTH)
◯ Allied Hospital
◯ Ali Medical Centre
◯ Alrazi Health Care Ait
◯ Alrazi Health Care Dha
◯ American Eye Centre
◯ Arif Memorial Hospital
◯ Asfaque Memorial Hospital
◯ Ayub Teaching Hospital
◯ Azmat Rashid
◯ Bahria International Hospital
◯ Baqai Institute Of Oncology
◯ Baqai University Hospital
◯ Bilal Hospital
◯ Burhani Hospital
◯ Cheema Hospital
◯ Chenab General Hospital
◯ Chiniot General Hospital
◯ City Hospital
◯ City Hospital
◯ City Hospital
◯ CMH Lahore
◯ Darul Sehat Hospital
◯ DHQ Sargodha
◯ Dr Anklesaria Eye Clinic & Hospital
◯ Dr. Akbar Niazi Teaching Hospital
◯ Excel Labs
◯ Faisal Hospital
◯ Faiz Rehman Hospital
◯ Family Hospital
◯ Farooq Hospital
◯ Farooq Hospital- West Wood Branch
◯ Fatima Memorial Hospital
◯ Fauji Foundation Hospital
◯ First Aid Orthopaedic Maternity General Hospital
◯ Gondal Medical Complex
◯ Goolbanoo & Dr Burjor Anklesaria Hos
◯ Habib Medical Centre
◯ Hafeez Eye Clinic
◯ Hamid Latif Hospital
◯ Haq Orthopedics
◯ Hashmani`S Hospital
◯ Hashmani`S Hospital
◯ Hassan General Hospital

◯ Hayatabad Medical Complex Peshawar
◯ Hilal-E-Ahmar Hospital
◯ Hira General Hospital
◯ Hope Medical Dental & Diagnostics
◯ Ibn-E-Seena Hospital Complex
◯ Iftikhar Memorial Hospital
◯ Imam Clinic
◯ Imam Zain Ul Abdin Hospital
◯ Iqra Medical Complex
◯ Islam Central Hospital
◯ Isra University Hospital
◯ Ittefaq Hospital
◯ Jinnah Hospital Lahore
◯ Karachi Adventist Hospital
◯ Khadija Poly Clinic
◯ Khyber Teaching Hospital Peshawar
◯ Laeeque Rafiq Hospital
◯ Lady Reading
◯ Lahore General Hospital
◯ Liaqat National Hospital
◯ Liaquat University Hospital Jamshoro
◯ Life Care Consultant Clinics
◯ Life Care Hospital
◯ Mamji Hospital
◯ Maroof International Hospital
◯ Maryam Memorial Hospital
◯ Mid City Hospital
◯ Mumtaz Bakhtawar Hospital Raiwind
◯ Murshid Hospital
◯ National Eye Center
◯ National Eye Center
◯ National Hospital & Medical Centre
◯ National Medical Centre Pvt Ltd
◯ Nehal Hospital
◯ Neurospinal & Cancer Care Institute
◯ Nishtar Medical Hospital
◯ Noor Hospital
◯ Nusrat Hospiatl
◯ OMC
◯ Pakistan Institue of Neurological Sciences
◯ Pakistan Railway Hospital
◯ Park Lane Hosptal
◯ Patel Hospital
◯ Peoples University Nawabshah
◯ PIC
◯ Prime Care Hospital
◯ Punjab Social Security Hospital Raiwind
◯ Quaid-E-Azam International Hospital
◯ Rai Medical College Teaching Hospital
◯ Rasheed Hospital
◯ Rasheed Hospital
◯ Rawal Institute Of Health Sciences
◯ Red Crescent Cardiac Hospital & C.C.U
◯ Red Crescent General Hospital & Ambulance Centre
◯ Remedial Hospital
◯ Rmi
◯ Saahil Hospital
◯ Sadiq Hospital
◯ Saifee Hospital Trust
◯ Saira Memorial Hospital
◯ Shalamar Hospital
◯ Shamsi Hospital
◯ Shaukat Khanum
◯ Shaukat Khanum ,Diagnostic Centre & Clinic
◯ Shifa International Hospital
◯ Sir Syed Trust Hospital
◯ Sughra Shafi Medical Complex (Sahara For Life Trust)
◯ Sultan Hospital
◯ Surgimed Hospital
◯ Surraya Azeem Hospital

	◯ Tabba Heart Institute<br>◯ Tabba Kidney Institute<br>◯ Umer Hospital<br>◯ Valley Medical Complex<br>◯ Yousaf Qureshi Hospital<br>◯ Ziauddin Hospital Clifton<br>◯ Ziauddin Hospital Kemari<br>◯ Ziauddin Hospital North Nazimabad<br>◯ Other
If other, please manually specify the hospital name	__________________________
Date of admission<br><br>Time should be entered in the 24-hour clock format.	__________________________
Date of discharge<br><br>Time should be entered in the 24-hour clock format.	__________________________
Procedure<br><br>The drop-down menu includes an extensive list of procedures. Please choose the most suitable code for the main procedure that the patient underwent. For example, if a patient underwent appendectomy and laparoscopic washout, please record this as appendectomy (the main procedure performed).	◯ Axilla - Axillary Clearance (Lymphadenectomy)<br>◯ Unilateral Mastectomy<br>◯ Bilateral Mastectomy<br>◯ Breast Reconstruction (Implant)<br>◯ Breast Reconstruction (Tissue Flap)<br>◯ Lumpectomy/ Wide Local Excision +/- Wire<br>◯ Coronary Artery Bypass Graft (CABG)<br>◯ Aortic Valve Surgery (Valvular)<br>◯ Mitral Valve Surgery (Valvular)<br>◯ Right Colectomy<br>◯ Low Anterior Resection of Colon<br>◯ Abdominoperineal Resection<br>◯ Craniotomy (Brain Tumors)<br>◯ Craniotomy (Aneurysms)<br>◯ Insertion of Ventriculoperitoneal Shunt<br>◯ Inguinal Hernia Repair<br>◯ Ventral Hernia Repair<br>◯ Cholecystectomy<br>◯ Exploratory Laparotomy<br>◯ Appendectomy<br>◯ Hysterectomy (Benign Indications)<br>◯ Hysterectomy (Uterine Malignancy)<br>◯ Caesarean Section<br>◯ Debulking Surgery (Ovarian Cancer)<br>◯ Myomectomy<br>◯ Total Knee Replacement (Arthroplasty)<br>◯ Total Hip Replacement (Arthroplasty)<br>◯ Bipolar Hemiarthroplasty<br>◯ Dynamic Hip Screw<br>◯ Laminectomy (Lumbar)<br>◯ Laminectomy (Cervical)<br>◯ Laminectomy (Thoracic)<br>◯ Microdiscectomy<br>◯ Limb Bypass Procedures<br>◯ Varicose Vein Repair<br>◯ Arteriovenous Fistula for Dialysis Axis<br>(Please select the most appropriate procedure)
Age<br><br>Please mention age of patient at the time of surgery.	__________________________<br>(in years)

Gender	◯ Male<br>◯ Female
Weight<br><br>Please mention the weight of the patient at the time of the surgery.	____________________<br>(in kilograms (kgs))
Height<br><br>Please mention the height of the patient at the time of surgery.	____________________<br>(in cm)
American Society of Anesthesiologists (ASA) classification<br><br>Full definitions	◯ Grade 1: Normal healthy person<br>◯ Grade 2: Mild systemic disease (e.g., controlled diabetes, hypertension)<br>◯ Grade 3: Severe systemic disease not incapacitating (e.g., moderate chronic obstructive pulmonary disease, malignancy, diabetes)<br>◯ Grade 4: Incapacitating systemic disease that is a constant threat to life (e.g., pre-eclampsia, heavy bleeding)<br>◯ Grade 5: Moribund patient, not expected to survive with or without operation (e.g., major trauma)<br>◯ Not recorded
Does the patient suffer from HIV/AIDS?	◯ Yes, on antiretroviral therapy<br>◯ Yes, not on antiretroviral therapy<br>◯ No<br>◯ Unknown
If yes, within these 12 months, what was the last CD4 count (cells per ml) of the patient?	____________________
Does the patient have diabetes mellitus?	◯ Yes, diet-controlled<br>◯ Yes, medication (non-insulin) controlled<br>◯ Yes, insulin controlled<br>◯ No, patient does not have diabetes
Was the patient taking oral or intravenous steroids preoperatively?<br><br>These do not include steroids administered topically (i.e., via inhalers, creams).	◯ Yes<br>◯ No<br>(Please only mark yes if the patient took steroids for 10 days or more in the 30 days prior to surgery)
Was the patient taking immunosuppressants preoperatively?<br><br>These can include drugs like methotrexate, azathioprine, mycophenolate mofetil, anti-TNF alpha antibodies.	◯ Yes<br>◯ No<br>(Please only mark yes if the patient took immunosuppressants for 10 days or more in the 30 days prior to surgery)
Was the patient receiving chemotherapy for cancer preoperatively?<br><br>This would include drugs like capecitabine, oxaliplatin, fluorouracil, etc., and not hormone-modifying drugs, i.e., tamoxifen, anastrozole or goserelin, etc.	◯ Yes<br>◯ No<br>(Please only mark yes if the patient received chemotherapy in the 30 days prior to surgery.)

What was the patient's smoking status at the time of surgery?	◯ Never smoked<br>◯ Ex-smoker (stopped in the 6 weeks prior to surgery)<br>◯ Ex-smoker (stopped ≥6 weeks prior to surgery)<br>◯ Current smoker (at the time of surgery)
Was the patient suffering from tuberculosis?	◯ Yes, diagnosed within 9 months of surgery<br>◯ Yes, diagnosed ≥9 months of surgery<br>◯ No, never diagnosed with tuberculosis
Please select how this patient was identified for inclusion	☐ Theatre logbook review<br>☐ From planned theatres lists or diaries (i.e., before the surgery had occurred)<br>☐ Handover lists<br>☐ Memory recall from staff<br>☐ Review of ward lists<br>(Select all that apply.)

Preoperative Form

Urgency of surgery?<br><br>An elective operation is one where it is planned prior to the patient's admission to hospital.<br><br>An emergency surgery is defined as any surgery during the same admission as diagnosis. Emergency surgeries may take place on the day of hospital admission or on any other day during hospital admission.	◯ Elective<br>◯ Emergency
Was the surgery performed as day-case surgery?<br><br>A day-case surgery is defined as surgery performed with length of stay < 24 hours (i.e., without any overnight hospital stay; surgical day care procedures).	◯ Yes<br>◯ No<br>(If a day-case surgery was planned, but the patient required hospital stay ≥24 hours, select "No".)
Indication for surgery?<br><br>Benign disease: Any disease/condition that is not related to trauma, malignancy, or obstetrics. These may include benign neoplastic or non-neoplastic conditions.<br><br>Malignancy: Suspected or confirmed malignancies.<br><br>Trauma: Any cause of injury, including burns.<br><br>Obstetric: Procedures related to childbirth, i.e., cesarean section, etc.	◯ Benign disease<br>◯ Malignancy<br>◯ Trauma<br>◯ Obstetric
If you selected malignancy, was this cancer surgery planned to be curative, palliative, or diagnostic?	◯ Curative<br>◯ Palliative<br>◯ Surgery for diagnostic purpose only
Patient preparation measures?	☐ Pre-op bath/ shower (full body)<br>☐ Antimicrobial soap used<br>☐ Plain soap used<br>☐ Others<br>(Please select all that apply)

If other:	__________________________
Was hair removed?	◯ Yes, razor used<br>◯ Yes, clippers used<br>◯ No
If yes, where was hair removed?	◯ Home<br>◯ Ward<br>◯ Theatre

Intraoperative Form

Which of the following skin preparation agents were used for surgical skin preparation?	☐ Chlorhex-alc<br>☐ Iodine-alc<br>☐ Chlorhex-aq<br>☐ Iodine-aq<br>(Select all that apply.)
Skin allowed to fully dry before incision?	◯ Yes<br>◯ No
Surgical hand preparation?	☐ Alcohol-based hand rub<br>☐ Antimicrobial soap + water<br>☐ Plain soap + water<br>(Select all that apply)
Headcount at start of operation<br><br>Please document number of individuals (excluding the patient) in the operating room at the time of knife-to-skin.	__________________________
Headcount at end of operation<br><br>Please document number of individuals (excluding the patient) in the operating room at the time of wound closure.	__________________________
Was the surgical site marked?	◯ Yes<br>◯ No
Was a time-out done before starting the surgery?<br><br>Time-out requires confirmation of patient and surgeon identity, site, procedure, and consent status<br><br>Further details can be viewed in the WHO Safe Surgery Checklist	◯ Yes<br>◯ No

Operative approach<br><br>Minimally invasive surgeries use specific instruments designed to reduce the invasiveness of the procedure. Examples may include completely laparoscopic, thoracoscopic, arthroscopic procedures, etc.<br><br>"Minimally invasive procedure converted to open" includes any procedure that was initially planned as a minimally invasive procedure but was switched to an open surgery intraoperatively.<br><br>"Hybrid" option should be selected when a minimally invasive approach is used for one body compartment (i.e., thorax) while an open approach is used for another body compartment (i.e., abdomen)	◯ Planned open surgery<br>◯ Planned and performed as minimally invasive surgery<br>◯ Minimally invasive surgery converted to open<br>◯ Hybrid surgery (i.e., laparoscopic abdomen, open chest)
Anesthesia?	☐ Local<br>☐ Nerve block<br>☐ Spinal<br>☐ Epidural<br>☐ General<br>(Select all that apply)
Was an epidural inserted during surgery for postoperative pain relief?<br><br>Use of a single shot of spinal anesthetic would not count as an epidural.	◯ Yes<br>◯ No<br>(Only answer "Yes" if an epidural catheter was inserted during surgery (even if inserted for a short time).)
Surgical wound class?<br><br>Would classifications are usually measured by operating surgeons and documented in records.<br><br><br>- Clean-contaminated: An incision through the respiratory, alimentary or genitourinary tract under controlled conditions with no direct contamination encountered.<br>- Contaminated: An operation where there is major break in sterile technique or gross spillage from the gastrointestinal tract, or an incision where acute non-purulent inflammation is encountered or where procedures involve traumatic wounds that have been open for between 12 hours and 24 hours.<br>- Dirty: An incision undertaken where viscera are perforated, where acute inflammation or necrosis is encountered, or where there is delayed operation on traumatic wounds.	◯ Clean: Sterile tissue with no resident bacteria<br>◯ Clean-contaminated: Controlled entry to tissue with resident bacteria<br>◯ Contaminated: Uncontrolled entry to tissue with bacteria<br>◯ Dirty/ infected: Heavy contamination (e.g., soil in wind) or infection already established
Antibiotic used?<br><br>This may include preoperative, intraoperative, and/or postoperative antibiotic use.	◯ Yes<br>◯ No
If yes, used for treatment before surgery (antibiotics within 24 hours of surgery)<br><br>If antibiotics are used within 24 hours prior to surgery, please select "Yes" and document duration of preoperative antibiotic use in days.	◯ Yes<br>◯ No<br>(If antibiotics are used within 24 hours prior to surgery, please select "Yes")

Please mention total days of preoperative antibiotic use	________________
If yes, used for prophylaxis at the point of incision (i.e., standard hospital prophylaxis)	◯ Yes<br>◯ No
If yes, continued at the end of surgery (i.e., extended prophylaxis after surgery)<br><br>If antibiotics are continued postoperatively, please select "Yes" and document duration of postoperative antibiotics used.	◯ Yes<br>◯ No
Please mention total days of postoperative antibiotic use	________________
Admission to procedure time<br><br>Please mention time duration from hospital admission till start of surgery (knife-to-skin).	________________<br>(in hours)
Time of operation start (time of knife-to-skin)?	________________<br>(Time should be entered in the 24-hour clock format.)
Time of operation end (when wound closed)?	________________<br>(Time should be entered in the 24-hour clock format.)
Length of operation (knife-to-skin until wound closure)?	________________<br>(In Hours)
Was a World Health Organization (or equivalent) surgical safety checklist used?	◯ Yes<br>◯ No, but available in this center<br>◯ No, not available in this center

Postoperative Form

Did the patient require intensive care unit (ICU) admission?	◯ Yes<br>◯ No
Duration of ICU stay	________________<br>(in hours)
Length of hospital stay	________________<br>(in days)
Was surgical site infection (SSI) seen in 30 days postoperatively?	◯ Yes<br>◯ No

How was the SSI detected?	◯ Inpatient (during the index hospital admission for surgery)<br>◯ At readmission<br>◯ At post-discharge outpatient clinic follow-up<br>◯ At post-discharge telephonic follow-up
Date of onset of SSI?<br><br>Please mention date when the patient started experiencing symptoms related to SSI. In case this date is unknown, mention date of diagnosis of SSI.	________________________
Criteria for SSI	☐ Abscess or other evidence of infection found during a re-operation, by radiology or histopathology examination<br>☐ Antibiotics prescribed by GP for SSI (patient reported only)<br>☐ Aspirated fluid/swab of surgical site yields organisms and pus cells are present<br>☐ Clinician's diagnosis<br>☐ Fever (temperature 38ºC or more)<br>☐ Heat<br>☐ Incision spontaneously dehisces or opened by surgeon/dehisces<br>☐ Localized pain or tenderness<br>☐ Localized swelling<br>☐ Purulent drainage<br>☐ Redness<br>(Select all that apply.)
SSI Type?	◯ Superficial incisional<br>◯ Deep incisional<br>◯ Organ/ space
If organ/space was detected, specific site of organ/space SSI?	☐ Arterial or venous<br>☐ Bone (osteomyelitis)<br>☐ Breast abscess/mastitis<br>☐ Endocardium<br>☐ Gastrointestinal tract<br>☐ Intra- abdominal<br>☐ Intracranial<br>☐ Joint or bursa<br>☐ Mediastinum<br>☐ Meningitis<br>☐ Myocardium or pericardium<br>☐ Vaginal cuff<br>☐ Vertebral disc space<br>(Select all that apply)
How was SSI treated?	☐ Operative drainage<br>☐ Wound opened outside of operating theatre<br>☐ Antibiotics<br>(Select all that apply.)
Was the patient readmitted due to surgical site infection?	◯ Yes<br>◯ No
If yes, date of SSI-related readmission	________________________

Was a wound swab sent for microbiological assessment?	◯ Yes<br>◯ No
What bacteria, if any, were identified?	☐ Gram-negative bacilli<br>☐ Staphylococcus aureus<br>☐ Anaerobe<br>☐ Pseudomonas species<br>☐ Coagulase-negative staphylococcus<br>☐ Enterococcus species<br>☐ Streptococcus species<br>☐ Candida species<br>☐ Other organism
Please specify the 'other' bacteria cultured	__________________________
Sensitivity profile of Gram Negative Bacilli cultured towards the antimicrobial prophylaxis used<br><br>This question refers to sensitivity and resistance of the causative organism(s) to the antibiotic used for prophylaxis	◯ Sensitive to antibiotic<br>◯ Resistant to antibiotic<br>◯ Sensitivity not tested
Sensitivity profile of Staphylococcus Aureus cultured towards the antimicrobial prophylaxis used<br><br>This question refers to sensitivity and resistance of the causative organism(s) to the antibiotic used for prophylaxis	◯ Sensitive to antibiotic<br>◯ Resistant to antibiotic<br>◯ Sensitivity not tested
Sensitivity profile of Anaerobe cultured towards the antimicrobial prophylaxis used<br><br>This question refers to sensitivity and resistance of the causative organism(s) to the antibiotic used for prophylaxis	◯ Sensitive to antibiotic<br>◯ Resistant to antibiotic<br>◯ Sensitivity not tested
Sensitivity profile of Pseudomonas cultured towards the antimicrobial prophylaxis used<br><br>This question refers to sensitivity and resistance of the causative organism(s) to the antibiotic used for prophylaxis	◯ Sensitive to antibiotic<br>◯ Resistant to antibiotic<br>◯ Sensitivity not tested
Sensitivity profile of Coagulase Negative Staphlococcus cultured towards the antimicrobial prophylaxis used<br><br>This question refers to sensitivity and resistance of the causative organism(s) to the antibiotic used for prophylaxis	◯ Sensitive to antibiotic<br>◯ Resistant to antibiotic<br>◯ Sensitivity not tested
Sensitivity profile of Enterococcus cultured towards the antimicrobial prophylaxis used<br><br>This question refers to sensitivity and resistance of the causative organism(s) to the antibiotic used for prophylaxis	◯ Sensitive to antibiotic<br>◯ Resistant to antibiotic<br>◯ Sensitivity not tested

Sensitivity profile of Streptococcus cultured towards the antimicrobial prophylaxis used<br><br>This question refers to sensitivity and resistance of the causative organism(s) to the antibiotic used for prophylaxis	◯ Sensitive to antibiotic<br>◯ Resistant to antibiotic<br>◯ Sensitivity not tested
Sensitivity profile of Candida Species cultured towards the antimicrobial prophylaxis used<br><br>This question refers to sensitivity and resistance of the causative organism(s) to the antibiotic used for prophylaxis	◯ Sensitive to antibiotic<br>◯ Resistant to antibiotic<br>◯ Sensitivity not tested
Sensitivity profile of Other Species cultured towards the antimicrobial prophylaxis used<br><br>This question refers to sensitivity and resistance of the causative organism(s) to the antibiotic used for prophylaxis	◯ Sensitive to antibiotic<br>◯ Resistant to antibiotic<br>◯ Sensitivity not tested
Was any other hospital-acquired infection seen within 30 days postoperatively?<br><br>Hospital-acquired infections can also be referred to as nosocomial infections.	☐ Yes, urinary tract infection<br>☐ Yes, pneumonia<br>☐ Yes, central venous line infection<br>☐ Yes, peripheral line infection<br>☐ Yes, other<br>☐ No<br>(Select all that apply)
If other, please specify	__________________________
Date of onset of Urinary Tract Infection?	__________________________
Date of onset of Pneumonia?	__________________________
Date of onset of Central Venous Line Infection?	__________________________
Date of onset of Peripheral Line Infection?	__________________________
Date of onset of other Infection?	__________________________
Mortality within 30 days of surgery	◯ Yes<br>◯ No
Date of death	__________________________
Was there any unexpected reintervention within 30 days postoperatively?<br><br>In a case where a plan is made at the time of the original operation for a "relook" surgery, this is not an "unexpected" reintervention.	☐ Yes, surgical<br>☐ Yes, endoscopic<br>☐ Yes, interventional radiology<br>☐ No

Date of unexpected surgical reintervention? ________________

Date of unexpected endoscopic reintervention? ________________

Date of unexpected interventional radiology reintervention? ________________

How was 30-day follow-up completed?

- ☐ Follow-up during the inpatient hospital stay
- ☐ Follow-up during hospital readmissions
- ☐ Post-discharge outpatient clinic follow-up
- ☐ Post-discharge telephonic follow-up
- ☐ Discharged before 30 days and not contacted again

(Please select all that apply)

Was telephone follow-up done at your hospital?

- ☐ Yes, at day 3
- ☐ Yes, at day 15
- ☐ Yes, at day 30
- ☐ No

(Please select all that apply)

Should this record be included in the analysis?

- ◯ Include, this record is a valid record for a patient who fulfils the inclusion criteria
- ◯ Exclude - patient does not fulfill inclusion criteria
- ◯ Exclude - patient did not undergo surgery (operation cancelled)
- ◯ Exclude - duplicate record
- ◯ Exclude - patient withdrew consent
- ◯ Exclude - test/practice record or record created by error
