# Supplementary material for: Epidemiology and risk factors of surgical site infections in elective surgeries in Pakistan (2022–2023): a multicentre, prospective cohort study from PakSurg 1
Source: Lancet Reg Health Southeast Asia. 2026 Jun 1;50:100786. doi: 10.1016/j.lansea.2026.100786 (PMC13253196; doi:10.1016/j.lansea.2026.100786)
Supplement: Supplementary Appendix 2 [file mmc2.docx]

**Epidemiology and Risk Factors of Surgical Site Infections in Elective Surgeries in Pakistan (2022-2023): A Multicentre, Prospective Cohort Study from PakSurg 1**

**Supplementary Appendix 2: Tables and Figures**

**Supplementary Figure 1:** PakSurg 1 Final Cohort Creation

**Supplementary Figure 2**: Graphical Summary of the PakSurg 1 Study Design and Key Findings

**Supplementary Table 1:** Individual Hospital Characteristics

**Supplementary Table 2:** Surgical Specialties Covered Per Institution

**Supplementary Table 3:** Average Number of Eligible Procedures Performed Per Month

**Supplementary Table 4:** Number of Surgical Faculty Operating Per Specialty

**Supplementary Table 5:** Number of Surgical Trainees Operating Per Specialty

**Supplementary Table 6**: Procedure Counts Included in Final Analysis

**Supplementary Table 7**: Variable Importance and Standardized Coefficients from Elastic Net Penalized Logistic Regression

**Supplementary Figure 1:** PakSurg 1 Final Cohort Creation

**
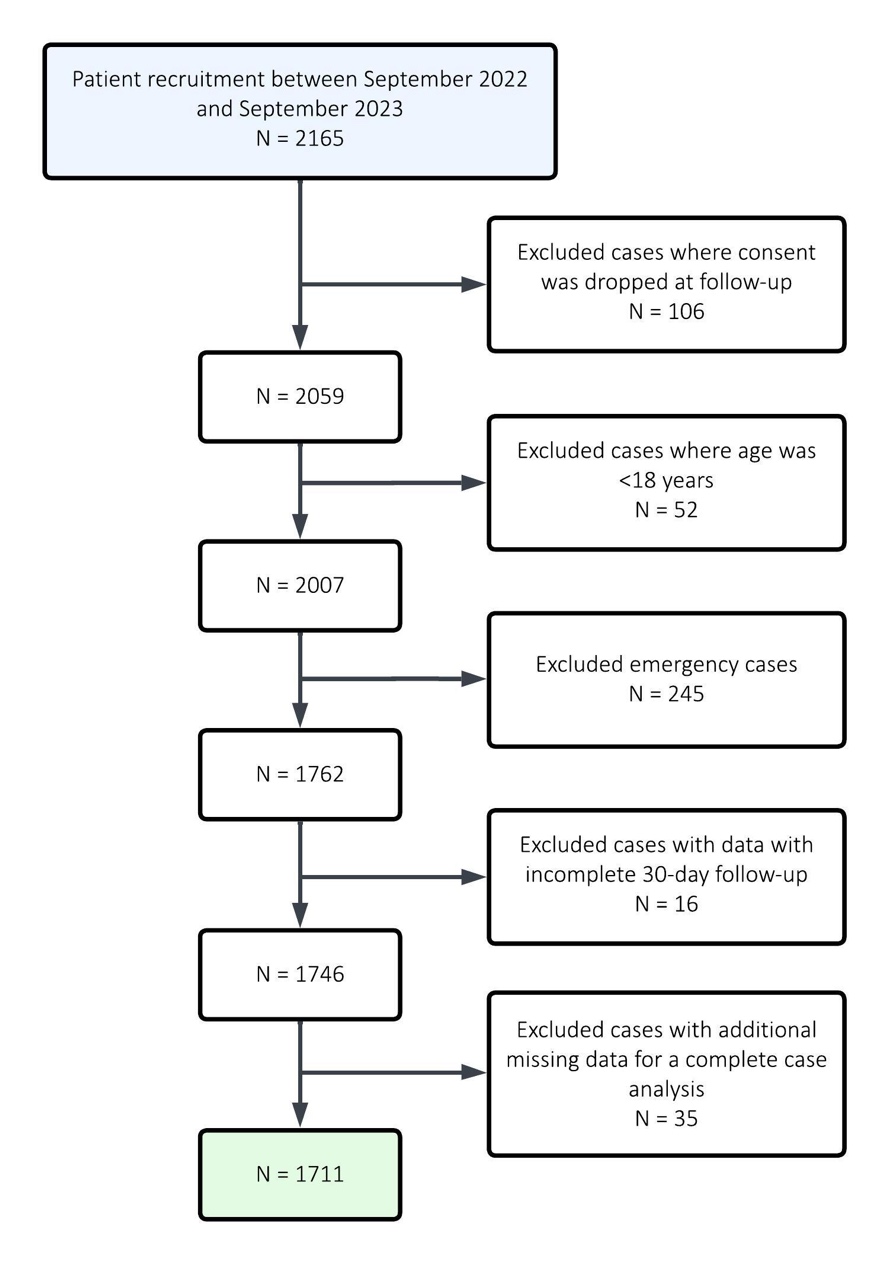
**

**Supplementary Figure 2**: Graphical Summary of the PakSurg 1 Study Design and Key Findings

*Caption:* *The figure illustrates the multicentre prospective cohort design, study population, follow-up period, overall incidence of surgical site infections SSI, and independent predictors identified in multivariable analysis. Upward arrows indicate factors associated with increased odds of SSI, and downward arrows indicate factors associated with reduced odds*

**
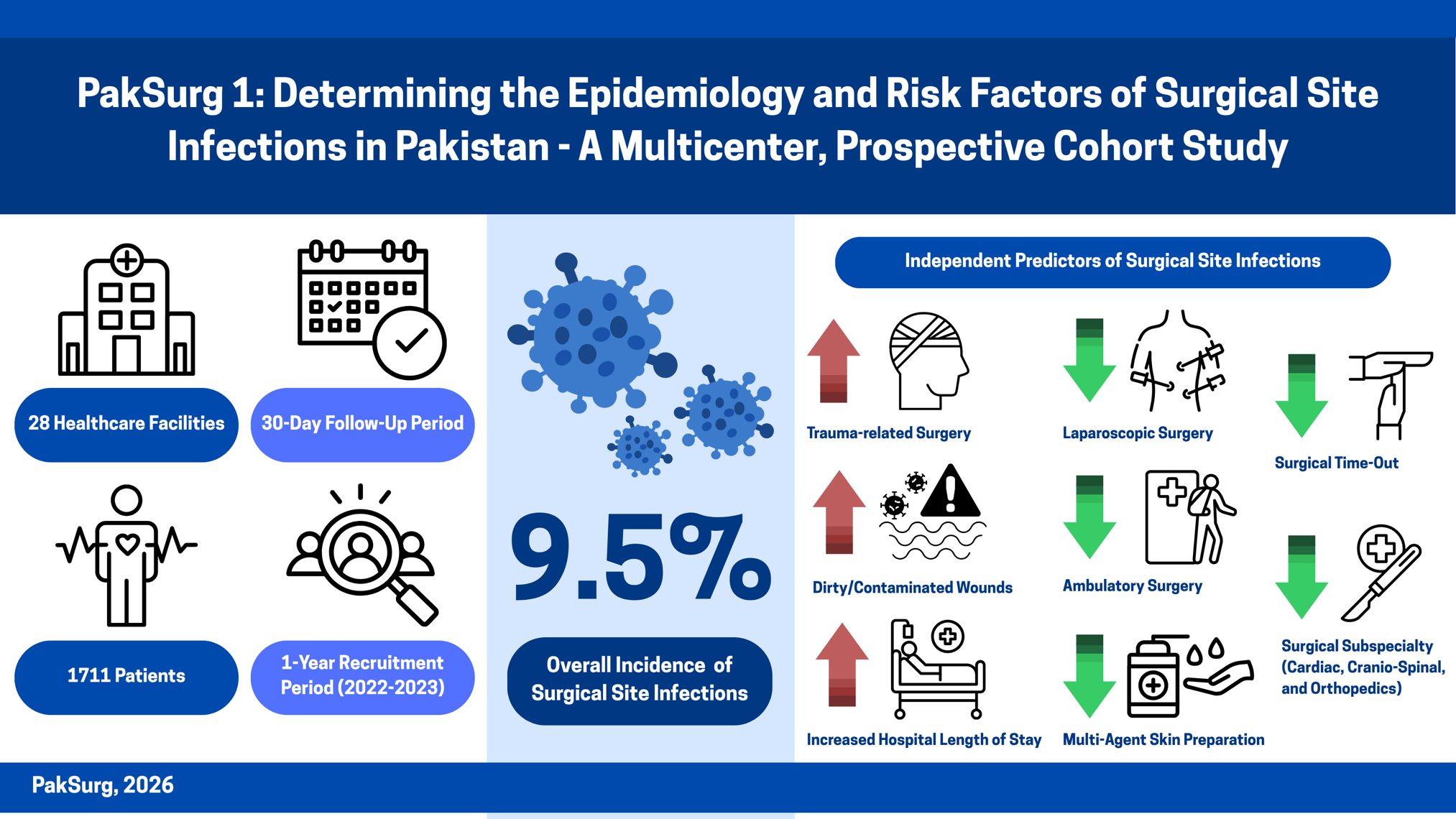
**

**Supplementary Table 1: Individual Hospital Characteristics**

| **No.** | **Institution** | **City** | **Service Level** | **Teaching Hospital** | **Category** | **Number of Beds** | **Cases Contributed** |
| --- | --- | --- | --- | --- | --- | --- | --- |
| 1 | Abbas Institute of Medical Sciences | Muzaffarabad | Tertiary | Yes | Public | 400 | 21 |
| 2 | Abbassi Shaheed Hospital | Karachi | Tertiary | Yes | Public | 850 | 96 |
| 3 | Aga Khan University Hospital | Karachi | Tertiary | Yes | Private | 710 | 340 |
| 4 | Allama Iqbal Memorial Teaching Hospital | Sialkot | Tertiary | Yes | Public | 650 | 16 |
| 5 | Allied Hospital | Faisalabad | Tertiary | Yes | Public | 1500 | 95 |
| 6 | Amina Hospital | Sialkot | Tertiary | No | Private | 50 | 19 |
| 7 | Ayub Teaching Hospital | Abbottabad | Tertiary | Yes | Public | 1400 | 75 |
| 8 | Bolan Medical Complex | Quetta | Tertiary | Yes | Public | 1200 | 30 |
| 9 | Combined Military Hospital (CMH), Lahore | Lahore | Tertiary | Yes | Public | 600 | 4 |
| 10 | Combined Military Hospital (CMH), Muzaffarabad | Muzaffarabad | Tertiary | Yes | Public | 400 | 20 |
| 11 | District Headquarters Teaching Hospital | Sargodha | Tertiary | Yes | Public | 730 | 48 |
| 12 | Dr. Akbar Niazi Teaching Hospital | Islamabad | Tertiary | Yes | Private | 500 | 105 |
| 13 | Fatima Memorial Hospital | Lahore | Tertiary | Yes | Private | 510 | 130 |
| 14 | Hayatabad Medical Complex | Peshawar | Tertiary | Yes | Public | 1280 | 110 |
| 15 | Holy Family Hospital | Islamabad | Tertiary | Yes | Public | 900 | 10 |
| 16 | Isra University Hospital | Hyderabad | Tertiary | Yes | Private | 600 | 100 |
| 17 | Jinnah Hospital | Lahore | Tertiary | Yes | Public | 1500 | 82 |
| 18 | Khyber Teaching Hospital | Peshawar | Tertiary | Yes | Public | 1600 | 30 |
| 19 | Lady Reading Hospital | Peshawar | Tertiary | Yes | Public | 2000 | 64 |
| 20 | Lahore General Hospital | Lahore | Tertiary | Yes | Public | 1600 | 4 |
| 21 | Liaquat University of Medical and Health Sciences | Jamshoro | Tertiary | Yes | Public | 700 | 36 |
| 22 | Lyari General Hospital | Karachi | Tertiary | Yes | Public | 150 | 55 |
| 23 | Nishtar Medical Hospital | Multan | Tertiary | Yes | Public | 1800 | 29 |
| 24 | Northwest General Hospital | Peshawar | Tertiary | Yes | Private | 500 | 8 |
| 25 | Pakistan Railway Hospital | Rawalpindi | Secondary | Yes | Public | 350 | 152 |
| 26 | Peoples University of Medical & Health Sciences for Women | Nawabshah | Tertiary | Yes | Public | 2000 | 26 |
| 27 | Punjab Institute of Neurosciences | Lahore | Tertiary | Yes | Public | 500 | 4 |
| 28 | Rai Medical College Teaching Hospital | Sargodha | Tertiary | Yes | Private | 500 | 2 |
| *Abbreviations: OBGYN: Obstetrics and Gynecology; CMH: Combined Military Hospital* | | | | | | | |

**Supplementary Table 2: Surgical Specialties and Number of Cases Covered Per Institution**

| **No.** | **Institution** | **Number of Cases Contributed** | | | | | | | | |
| --- | --- | --- | --- | --- | --- | --- | --- | --- | --- | --- |
|  |  | **Breast** | **Cardiac** | **Colorectal** | **Cranial** | **General** | **OBGYN** | **Orthopedics** | **Spinal** | **Vascular** |
| 1 | Abbas Institute of Medical Sciences | 1 |  |  |  | 10 | 10 |  |  |  |
| 2 | Abbassi Shaheed Hospital |  |  | 1 |  | 49 | 46 |  |  |  |
| 3 | Aga Khan University Hospital | 45 | 51 |  | 6 | 106 | 46 | 49 | 16 | 21 |
| 4 | Allama Iqbal Memorial Teaching Hospital | 4 |  |  |  | 8 | 4 |  |  |  |
| 5 | Allied Hospital | 13 |  | 4 | 6 | 41 | 3 | 9 | 17 | 2 |
| 6 | Amina Hospital |  |  |  |  | 19 |  |  |  |  |
| 7 | Ayub Teaching Hospital |  |  |  |  | 37 | 34 | 4 |  |  |
| 8 | Bolan Medical Complex |  |  |  |  | 29 | 1 |  |  |  |
| 9 | Combined Military Hospital (CMH), Lahore |  |  |  |  | 3 | 1 |  |  |  |
| 10 | Combined Military Hospital (CMH), Muzaffarabad |  |  |  |  | 7 | 13 |  |  |  |
| 11 | District Headquarters Teaching Hospital |  |  |  |  | 24 | 24 |  |  |  |
| 12 | Dr. Akbar Niazi Teaching Hospital | 6 |  |  | 10 | 62 | 24 | 3 |  |  |
| 13 | Fatima Memorial Hospital |  |  |  |  | 54 | 76 |  |  |  |
| 14 | Hayatabad Medical Complex |  | 43 |  |  | 44 | 1 | 17 | 4 | 1 |
| 15 | Holy Family Hospital |  |  |  |  |  | 10 |  |  |  |
| 16 | Isra University Hospital |  |  | 1 |  | 23 | 62 | 14 |  |  |
| 17 | Jinnah Hospital | 3 | 13 |  | 17 | 11 | 19 | 10 | 9 |  |
| 18 | Khyber Teaching Hospital |  |  | 2 | 3 |  | 18 | 7 |  |  |
| 19 | Lady Reading Hospital | 3 |  | 1 | 3 | 34 | 23 |  |  |  |
| 20 | Lahore General Hospital |  |  | 4 |  |  |  |  |  |  |
| 21 | Liaquat University of Medical and Health Sciences | 5 |  |  | 4 | 15 | 7 |  | 5 |  |
| 22 | Lyari General Hospital |  |  |  |  | 55 |  |  |  |  |
| 23 | Nishtar Medical Hospital | 3 |  |  | 4 |  | 15 |  | 7 |  |
| 24 | Northwest General Hospital |  |  |  | 2 | 2 |  |  | 4 |  |
| 25 | Pakistan Railway Hospital |  | 1 | 2 | 1 | 97 | 33 | 17 | 1 |  |
| 26 | Peoples University of Medical & Health Sciences for Women |  |  |  | 2 | 22 | 1 |  | 1 |  |
| 27 | Punjab Institute of Neurosciences |  |  |  |  |  |  |  | 4 |  |
| 28 | Rai Medical College Teaching Hospital |  |  |  |  |  | 2 |  |  |  |
| *Abbreviations: OBGYN: Obstetrics and Gynecology; CMH: Combined Military Hospital* | | | | | | | | | | |

**Supplementary Table 3: Average Number of Eligible Procedures Performed Per Month**

| **No.** | **Institution** | **Average Number of Eligible Procedures Performed Per Month** | | | | | | | | |
| --- | --- | --- | --- | --- | --- | --- | --- | --- | --- | --- |
|  |  | **Breast** | **Cardiac** | **Colorectal** | **Cranial** | **General** | **OBGYN** | **Orthopedics** | **Spinal** | **Vascular** |
| 1 | Abbas Institute of Medical Sciences | < 10 |  |  |  | 50-69 | >70 |  |  | 10-29 |
| 2 | Abbassi Shaheed Hospital |  |  |  |  |  | 10-29 |  |  |  |
| 3 | Aga Khan University Hospital | 10-29 | >70 |  | >70 | >70 | >70 | >70 | 30-49 | >70 |
| 4 | Allama Iqbal Memorial Teaching Hospital | 10-29 |  |  |  | 30-49 | >70 |  |  |  |
| 5 | Allied Hospital | 10-29 |  | 10-29 | 30-49 | >70 | 50-69 | 30-49 | 10-29 | < 10 |
| 6 | Amina Hospital |  |  |  |  | 30-49 |  |  |  |  |
| 7 | Ayub Teaching Hospital |  |  |  |  | >70 | >70 | 10-29 |  |  |
| 8 | Bolan Medical Complex |  |  |  |  | >70 | >70 |  |  |  |
| 9 | Combined Military Hospital (CMH), Lahore |  |  |  |  | 50-69 | 50-69 |  |  |  |
| 10 | Combined Military Hospital (CMH), Muzaffarabad |  |  |  |  | 30-49 | 50-69 |  |  |  |
| 11 | District Headquarters Teaching Hospital |  |  |  |  | 10-29 | 10-29 |  |  |  |
| 12 | Dr. Akbar Niazi Teaching Hospital | < 10 |  |  | < 10 | 10-29 | 10-29 | < 10 |  |  |
| 13 | Fatima Memorial Hospital |  |  |  |  | 50-69 | >70 |  |  |  |
| 14 | Hayatabad Medical Complex |  | 30-49 |  |  | >70 | >70 | 50-69 | >70 | 10-29 |
| 15 | Holy Family Hospital |  |  |  |  |  |  |  |  |  |
| 16 | Isra University Hospital |  |  | < 10 |  | 10-29 | 50-69 | < 10 |  |  |
| 17 | Jinnah Hospital | 30-49 | < 10 |  | 10-29 | 50-69 | 30-49 | 10-29 | 10-29 |  |
| 18 | Khyber Teaching Hospital |  |  | 10-29 | 10-29 |  | 30-49 | 10-29 |  |  |
| 19 | Lady Reading Hospital |  |  | >70 | >70 | >70 | >70 |  |  |  |
| 20 | Lahore General Hospital |  |  | 50-69 |  |  |  |  |  |  |
| 21 | Liaquat University of Medical and Health Sciences | 10-29 |  |  | < 10 | 30-49 | 50-69 |  | 10-29 |  |
| 22 | Lyari General Hospital |  |  |  |  | 10-29 |  |  |  |  |
| 23 | Nishtar Medical Hospital | < 10 |  |  | < 10 |  | 30-49 |  | < 10 |  |
| 24 | Northwest General Hospital |  |  |  | 30-49 | 30-49 |  |  | 10-29 |  |
| 25 | Pakistan Railway Hospital |  | < 10 | < 10 |  | 10-29 | 30-49 | 10-29 | 10-29 |  |
| 26 | Peoples University of Medical & Health Sciences for Women |  |  |  |  | 30-49 | 50-69 |  | 10-29 |  |
| 27 | Punjab Institute of Neurosciences |  |  |  |  |  |  |  | 30-49 |  |
| 28 | Rai Medical College Teaching Hospital |  |  |  |  |  | 10-29 |  |  |  |
| **Note:** Institutional data for a given subspecialty has only been reported if an institution contributed cases of that subspecialty to the PakSurg dataset.  *Abbreviations: OBGYN: Obstetrics and Gynecology; CMH: Combined Military Hospital* | | | | | | | | | | |

**Supplementary Table 4: Number of Surgical Faculty Operating Per Specialty**

| **No.** | **Institution** | **Number of Surgical Faculty Operating** | | | | | | | | |
| --- | --- | --- | --- | --- | --- | --- | --- | --- | --- | --- |
|  |  | **Breast** | **Cardiac** | **Colorectal** | **Cranial** | **General** | **OBGYN** | **Orthopedics** | **Spinal** | **Vascular** |
| 1 | Abbas Institute of Medical Sciences | 4 |  |  |  | 4 | 5 |  |  |  |
| 2 | Abbassi Shaheed Hospital |  |  |  |  |  | 10 |  |  |  |
| 3 | Aga Khan University Hospital | 3 | 6 |  | 8 | 11 | 28 | 12 | 8 | 3 |
| 4 | Allama Iqbal Memorial Teaching Hospital | 5 |  |  |  | 8 | 4 |  |  |  |
| 5 | Allied Hospital | 10 |  | 10 | 5 | 10 | 12 | 6 | 4 | 5 |
| 6 | Amina Hospital |  |  |  |  | 4 |  |  |  |  |
| 7 | Ayub Teaching Hospital |  |  |  |  | 10 | 15 | 5 |  |  |
| 8 | Bolan Medical Complex |  |  |  |  | 14 | 14 |  |  |  |
| 9 | Combined Military Hospital (CMH), Lahore |  |  |  |  | 4 | 7 |  |  |  |
| 10 | Combined Military Hospital (CMH), Muzaffarabad |  |  |  |  | 5 | 6 |  |  |  |
| 11 | District Headquarters Teaching Hospital |  |  |  |  | 4 | 5 |  |  |  |
| 12 | Dr. Akbar Niazi Teaching Hospital | 1 |  |  | 1 | 2 | 3 | 1 |  |  |
| 13 | Fatima Memorial Hospital |  |  |  |  | 7 | 8 |  |  |  |
| 14 | Hayatabad Medical Complex |  | 2 |  |  | 5 | 6 | 4 | 3 | N/A |
| 15 | Holy Family Hospital |  |  |  |  |  |  |  |  |  |
| 16 | Isra University Hospital |  |  | 3 |  | 7 | 12 | 2 |  |  |
| 17 | Jinnah Hospital | 8 | 1 |  | 5 | 25 | 12 | 12 | 5 |  |
| 18 | Khyber Teaching Hospital |  |  | 4 | 3 |  | 10 | 9 |  |  |
| 19 | Lady Reading Hospital | N/A |  | 4 | 5 | 12 | 12 |  |  |  |
| 20 | Lahore General Hospital |  |  | 6 |  |  |  |  |  |  |
| 21 | Liaquat University of Medical and Health Sciences | 7 |  |  | 2 | 12 | 4 |  | 3 |  |
| 22 | Lyari General Hospital |  |  |  |  | 2 |  |  |  |  |
| 23 | Nishtar Medical Hospital | 8 |  |  | 3 |  | 8 |  | 3 |  |
| 24 | Northwest General Hospital |  |  |  | 3 | 3 |  |  | 3 |  |
| 25 | Pakistan Railway Hospital |  | 1 | 2 |  | 3 | 3 | 2 | N/A |  |
| 26 | Peoples University of Medical & Health Sciences for Women |  |  |  |  | 30 | 50 |  | N/A |  |
| 27 | Punjab Institute of Neurosciences |  |  |  |  |  |  |  | 25 |  |
| 28 | Rai Medical College Teaching Hospital |  |  |  |  |  | 4 |  |  |  |
| **Note:** Institutional data for a given subspecialty has only been reported if an institution contributed cases of that subspecialty to the PakSurg dataset.  *Abbreviations: OBGYN: Obstetrics and Gynecology; CMH: Combined Military Hospital* | | | | | | | | | | |

**Supplementary Table 5: Number of Surgical Trainees Operating Per Specialty**

| **No.** | **Institution** | **Number of Surgical Trainees Operating** | | | | | | | | |
| --- | --- | --- | --- | --- | --- | --- | --- | --- | --- | --- |
|  |  | **Breast** | **Cardiac** | **Colorectal** | **Cranial** | **General** | **OBGYN** | **Orthopedics** | **Spinal** | **Vascular** |
| 1 | Abbas Institute of Medical Sciences | 5 |  |  |  | 8 | 5 |  |  |  |
| 2 | Abbassi Shaheed Hospital |  |  |  |  | N/A | 25 |  |  |  |
| 3 | Aga Khan University Hospital | 5 | 7 |  | 16 | 20 | 27 | 20 | 16 | 20 |
| 4 | Allama Iqbal Memorial Teaching Hospital | 6 |  |  |  | 12 | 18 |  |  |  |
| 5 | Allied Hospital | 25 |  | 25 | 13 | 25 | 25 | 15 | 12 | 16 |
| 6 | Amina Hospital |  |  |  |  | 0 |  |  |  |  |
| 7 | Ayub Teaching Hospital |  |  |  |  | 45 | 45 | 15 |  |  |
| 8 | Bolan Medical Complex |  |  |  |  | 40 | 25 |  |  |  |
| 9 | Combined Military Hospital (CMH), Lahore |  |  |  |  | 10 | 15 |  |  |  |
| 10 | Combined Military Hospital (CMH), Muzaffarabad |  |  |  |  | 11 | 12 |  |  |  |
| 11 | District Headquarters Teaching Hospital |  |  |  |  | 10 | 10 |  |  |  |
| 12 | Dr. Akbar Niazi Teaching Hospital | 1 |  |  | 1 | 8 | 8 | 2 |  |  |
| 13 | Fatima Memorial Hospital |  |  |  |  | 20 | 20 |  |  |  |
| 14 | Hayatabad Medical Complex |  | 6 |  |  | 20 | 25 | 15 | 10 | N/A |
| 15 | Holy Family Hospital |  |  |  |  |  | 7 |  |  |  |
| 16 | Isra University Hospital |  |  | 2 |  | 3 | 18 | 1 |  |  |
| 17 | Jinnah Hospital | 12 | 7 |  | 13 | 51 | 35 | 25 | 14 |  |
| 18 | Khyber Teaching Hospital |  |  | 25 | 15 |  | 40 | 25 |  |  |
| 19 | Lady Reading Hospital | N/A |  | 14 | 16 | 25 | 21 |  |  |  |
| 20 | Lahore General Hospital |  |  | 12 |  |  |  |  |  |  |
| 21 | Liaquat University of Medical and Health Sciences | 4 |  |  | 1 | 8 | 8 |  | 1 |  |
| 22 | Lyari General Hospital |  |  |  |  | 4 |  |  |  |  |
| 23 | Nishtar Medical Hospital | 10 |  |  | 8 |  | 12 |  | 8 |  |
| 24 | Northwest General Hospital |  |  |  | 10 | 10 |  |  | 8 |  |
| 25 | Pakistan Railway Hospital |  | 1 | 1 |  | 4 | 5 | 1 |  |  |
| 26 | Peoples University of Medical & Health Sciences for Women |  |  |  |  | 35 | 60 |  |  |  |
| 27 | Punjab Institute of Neurosciences |  |  |  |  |  |  |  | 10 |  |
| 28 | Rai Medical College Teaching Hospital |  |  |  |  |  | 2 |  |  |  |
| **Note:** Institutional data for a given subspecialty has only been reported if an institution contributed cases of that subspecialty to the PakSurg dataset.  *Abbreviations: OBGYN: Obstetrics and Gynecology; CMH: Combined Military Hospital* | | | | | | | | | | |

**Supplementary Table 6: Procedure Counts Included in Final Analysis**

| **Procedure** | **Count** |
| --- | --- |
| Aortic Valve Surgery (Valvular) | 11 |
| Appendectomy | 46 |
| Arteriovenous Fistula for Dialysis Axis | 16 |
| Axilla - Axillary Clearance (Lymphadenectomy) | 12 |
| Bilateral Mastectomy | 8 |
| Bipolar Hemiarthroplasty | 17 |
| Breast Reconstruction (Tissue Flap) | 1 |
| Caesarean Section | 286 |
| Cholecystectomy | 410 |
| Coronary Artery Bypass Graft (CABG) | 80 |
| Craniotomy (Aneurysms) | 5 |
| Craniotomy (Brain Tumors) | 39 |
| Debulking Surgery (Ovarian Cancer) | 8 |
| Dynamic Hip Screw | 50 |
| Exploratory Laparotomy | 68 |
| Hysterectomy (Benign Indications) | 131 |
| Hysterectomy (Uterine Malignancy) | 18 |
| Inguinal Hernia Repair | 142 |
| Insertion of Ventriculoperitoneal Shunt | 6 |
| Laminectomy (Cervical) | 4 |
| Laminectomy (Lumbar) | 54 |
| Laminectomy (Thoracic) | 3 |
| Limb Bypass Procedures | 1 |
| Low Anterior Resection of Colon | 5 |
| Lumpectomy/ Wide Local Excision +/- Wire | 21 |
| Microdiscectomy | 11 |
| Mitral Valve Surgery (Valvular) | 17 |
| Myomectomy | 26 |
| Right Colectomy | 8 |
| Total Hip Replacement (Arthroplasty) | 23 |
| Total Knee Replacement (Arthroplasty) | 47 |
| Unilateral Mastectomy | 50 |
| Varicose Vein Repair | 7 |
| Ventral Hernia Repair | 80 |

**Supplementary Table 7: Variable Importance and Standardized Coefficients from Elastic Net Penalized Logistic Regression**

*Model A (Including LOS: AUC=0.677*

| **Variable Rank** | **Predictor** | **Coefficient** |
| --- | --- | --- |
| 1 | Skin prep: Chlorhexidine (vs Both) | **+0.52** |
| 2 | Specialty: General Surgery (vs Cardiac) | **+0.48** |
| 3 | Time-out performed: Yes (vs No) | **−0.46** |
| 4 | Wound class: Contaminated/Dirty (vs Clean) | **+0.44** |
| 5 | Pre-op chemotherapy: Yes | **+0.38** |
| 6 | Specialty: OBGYN (vs Cardiac) | +0.35 |
| 7 | Immunosuppressant use: Yes | +0.32 |
| 8 | Skin prep: Povidone-Iodine (vs Both) | +0.30 |
| 9 | Skin fully dried: Yes | −0.29 |
| 10 | Planned open surgery (vs Hybrid) | +0.27 |
| 11 | Hospital LOS (days) | +0.25 |
| 12 | Surgical site marked: Yes | −0.23 |

*Model B (Excluding LOS): AUC=0.650*

| **Variable Rank** | **Predictor** | **Coefficient** |
| --- | --- | --- |
| 1 | Skin prep: Chlorhexidine (vs Both) | **+0.50** |
| 2 | Wound class: Contaminated/Dirty (vs Clean) | **+0.47** |
| 3 | Time-out performed: Yes | **−0.45** |
| 4 | Pre-op chemotherapy: Yes | +0.39 |
| 5 | Specialty: General Surgery | +0.36 |
| 6 | Planned open surgery | +0.33 |
| 7 | Skin fully dried: Yes | −0.31 |
| 8 | Immunosuppressant use | +0.29 |
| 9 | Skin prep: Povidone-Iodine | +0.28 |
| 10 | Male sex | −0.26 |
| 11 | Surgical site marked | −0.24 |
| 12 | BMI ≥30 kg/m² | +0.21 |
