# Supplementary material for: Epidemiology and risk factors of surgical site infections in elective surgeries in Pakistan (2022–2023): a multicentre, prospective cohort study from PakSurg 1
Source: Lancet Reg Health Southeast Asia. 2026 Jun 1;50:100786. doi: 10.1016/j.lansea.2026.100786 (PMC13253196; doi:10.1016/j.lansea.2026.100786)
Supplement: Supplementary Appendix 3 [file mmc3.docx]

**Epidemiology and Risk Factors of Surgical Site Infections in Elective Surgeries in Pakistan (2022-2023): A Multicentre, Prospective Cohort Study from PakSurg 1**

**Supplementary Appendix 3: PakSurg Authorship List**

**Central Steering Committee (Aga Khan University Hospital, Karachi)**

**Leads:** Usama Waqar*, Shaheer Ahmed**

**Writing & Analysis Team:** Asad Saulat Fatimi***, Warda Ahmed***, Russell Seth Martins, Haseeb Waheed, Illiyun Banani, Dahir Ashfaq, Asma Altaf Merchant, Ronika Devi Ukrani, Manzar Abbas, Muskan Abdul Qadir.

**Outreach Team:** Izza Tahir***, Daniyal Ali Khan***, Hareem Rauf, Mahnoor Javaid, Sarim Raheel.

**Operations Team:** Muhammad Uzair***, Muhammad Umar Mahar***, Sehar Salim Virani, Faiqa Binte Aamir, Hamna Ganny, Mabrooka Kazi.

**Dissemination Team:** Hajra Arshad***, Muhammad Abbas Raza, Muhammad Jawad Amin Malik, Muhammad Ozair Awan.

**Faculty:** Abida Khalil Sattar, Amna Urooba, Aliya Begum Aziz, Erum Baig, Maheen Mansoor, Hina Inam, Mashal Shah, Muhammad Shahzad Shamim, Nadeem Siddiqui, Rehana Siddiqui, Sadaf Khan, Shahryar Noordin, Tabish Chawla, Syed Ather Enam****.

_________________________________________________________________

* Steering Committee Lead

** Steering Committee Associate Lead

*** Team lead

**** Principal Investigator

**Hospital Collaborators**

*In this section, hospital leads are indicated using the following*: ****

_________________________________________________________________

**Abbas Institute of Medical Sciences, Muzaffarabad:** Laiba Kiani**, Naeem Ahmed**, Shafaq Hanif, Mahnoor Zahra, Amna Aslam, Haasin Iqbal, Zarish Shuja, Huzaifa Shabbir, Umm-e-Roman, Areeba Kabeer, Amerzish Shahid, Aqsa Haider, Fatima Tanveer, Marriam Nazakat, Alveena Waheed, Sana Umer Kiani, Mehrish Batool, Sana Kokab, Syed Nazar Sherazi.

**Abbassi Shaheed Hospital, Karachi:** Mifrah Rahat Khan Sherwani**, Gulnaz Khalid**, Muhammad Tahir, Aisha Khatoon, Zainab Farooq, Shanza Gul, Hira Islam, Areeba Saleem, Aariz Hussain, Wajiha Shaikh, Areeba Fareed, Warisha Kanwal, Vousqa Zubair Ahmed, Aqsa Fareed, Fareeha, Sana Iqbal, Zoha Haq, Kanwar Arham, Abdul Haseeb, Anum Khalid, Iqra, Tazmeen Sabooh, Hania Arshad, Faiza Saleem, Tehreem, Fariya Majid, Sibqun Irfan, Abdul Ghafoor Qureshi, Moaaz Syed Nezami, Farhan Ahmed.

**Aga Khan University Hospital, Karachi:** Warda Ahmed**, Syed Ather Enam**, Tabish Chawla, Shahryar Noordin, Muhammad Shahzad Shamim, Sadaf Khan, Aliya Begum Aziz, Abida Khalil Sattar, Hina Inam, Nadeem Siddiqui, Madeeha Ali, Muhammad Taha Nasim, Umer Adnan, Bilal Lodhi, Muhammad Tabish Nasim, Linta Khan, Khushi Saleem, Muneeb Khalid, Mannal Ahmed, Muhammad Ibrahim, Maryam Shaukat, Faiza Qureshi, Sneha Bheesham, Zain Javed, Taha Shaikh, Syeda Samnita Batool Zaidi, Aqsa Amjad, Huzaifa Ahmed, Fatima Abdullah, Fiza Adnan Khan, Sibgha Alam, Konain Imran, Reyan Hussain Shaikh, Muneeb Ahmed, Saba Bilal Qamar, Abdul Hadi Shahid, Sana Farhan, Hania Fatima, Arshia Jahangir, Umme Abeeha Zafar, Wamiq Ali Shaikh, Muhammad Hyedar Anwar, Muhammad Abdullah Jamil, Hashim Ishfaq, Hufriya Mondegarian, Mohammad Zakriya, Varisha Madni, Mohammad Shahmeer Chaudhry, Hamza Haider, Hunaina Abid, Komal Fida Ali, Ismail Khan, Khadija Awais Sumra, Amna Irfan Ansari, Misbah Jahangir, Aliha Shabbir, Sheza Saqib, Ahmad Jan Anab, Eisha Saadat, Aiman Sultan, Yusra Imran, Talal Bin Tariq, Kinza Jawed, Dahir Ashfaq, Umair Saleem, Kashmala Hussain, Musa Salar, Hashim Salar, Hassan Khan Niazi, Sajjan Raja, Zayan Alidina, Mashal Waqas, Fiza Sohail Gagai, Abia Abdullah, Shiza Atif, Karishma Wali, Zainab Haider Ejaz, Maha Muzzamil Chaipiwala, Shilpa Golani, Alizeh Sonia Fatimi, Shalni Golani, Abdullah Ahmed, Khadijah Aslam, Maheen Qureshi, Muhammad Daniyal, Eman Anwar, Shahier Paracha, Salaar Ahmed, Shahzil Abdur Rehman Malik.

**Allama Iqbal Memorial Teaching Hospital, Sialkot:** Ayesha Nawal**, Muhammad Qasim Butt**, Muhammad Luqman Kaleem, Abdur Rehman, Nabiha Syed, Muhammad Waleed Ajmal.

**Allied Hospital, Faisalabad:** Unaiza Ahmad**, Muhammad Saleem Iqbal**, Nazar Hussain, Ammara Niaz, Muhammad Shoaib Bin Shakeel, Azka Irfan, Mahrukh Atif, Mahnoor Naeem, Noor Azhar, Labeeba Abdul Ghafoor, Momna Yousaf, Muhammad Bilal Ashraf, Ajia Ali Khan, Sumayya Sajid, Muhammad Ahmad Arsal, Abdul Moeid, Abdullah Nasir, Mashal Fatima Farooq, Syed Samar Ali Shah, Ahmad Hassan Gul, Irtaza Shafqat, Hafiz Muhammad Usama Zuhair, Umer Hussain, Muhammad Ali, Sarmad Naeem, Ahmad Butt, Kiran Fatima, Rameen Fatima, Maryam Haroon, Muhammad Usman Javed, Muhammad Huzaifa Khan, Muhammad Awais Ali, Sameer Ashraf, Bilal Habib, Ali Shehzad, Ammara Basit, Nahal Irshad, Abrar Ahmad, Ayyaz Ahmad, Syed Sheraz Ashiq, Mubeen Ahmad Randhawa, Adnan Ansar Sukhyra, Musharraf Ur Rehman, Ahsan Ali Akbar, Muhammad Zain Munir, Ghazi Umair Ahmad, Muhammad Zaman.

**Amina Hospital, Sialkot:** Shah Ahmed Cheema**, Nadeem Ahmed**.

**Ayub Teaching Hospital, Abbottabad:** Muhammad Iltaf**, Ruqqia Sultana**, Fazli Junaid, Wajeeha Khurshid, Muhammad Qaisar Shah, Jamal Ali, Sakhawat Ali, Salman Ahmad Khan, Muhammad Raza.

**Bolan Medical Complex, Quetta:** Zubair Ahmed**, Shakeel Akbar**, Saiqa Bazai, Abdifitah Bashir Omer, Fouzia Ali.

**Combined Military Hospital, Lahore:** Hunniya Bint-e-Riaz**, Muhammad Akmal**, Muhammad Imran, Ismail Mazhar, Mir Muhammad Rai, Rao Muhammad Waleed.

**Combined Military Hospital, Muzaffarabad:** Maaela Khan**, Amir Iqbal Ali**, Maryam Zubair, Arooj Sabahat, Talha Adil, Zoha Khan, Rehnaz Riaz, Poshmal Zahid, Arwah Asif.

**District Head Quarter Hospital, Sargodha:** Muhammad Qaisar Karim**, Afroza Abbas**, Ahmad Hassan Khan, Sufian Muhammad Zahid, Muhammad Muneer Haider, Muhammad Hassan Javeed, Areeba Mariam Mahmood, Muhammad Arsalan, Nabeel Akhtar.

**Dr. Akbar Niazi Teaching Hospital, Islamabad:** Saad Maqbool**, Ahmed Raza**, Munazzah Aziz, Muhammad Assad Javed, Bushra Kant, Muhammad Hanif, Muhammad Khurram Sajjad, Sarah Ishtiyaq, Zainab Shahid, Sidra Javaid, Areej Taswar, Fatima Batool, Syeda Minahal Kazmi, Sana Razzaq, Uroba Arshad, Amal Khan, Mahad Ali Khan, Arrham Hai, Syeda Zohva Zainub, Maham Farooq, Shehryar Javed, Muhammad Asher Javed, Anam Zafar, Eisha Faheem, Saliha Maqsood, Shaiza Jabbar, Noor Ul Zuha, Soma Siddique, Zubaria Qureshi, Muhammad Ali, Izah Sadiq, Amna Shahid.

**Fatima Memorial Hospital, Lahore:** Rimsha Zahid**, Khaleeq Ur Rehman**, Javed Shakir, Andleeb Kanwal, Noor Fatima, Hamna Zaman, Amun Mustafa, Mohammad Ahmad Farooq, Fizza Mansoor, Irtiqua Zaheer, Maham Murtaza, Sameen Fatima Zafar, Hassan Sarwar, Muhammad Umair Amjad.

**Hayatabad Medical Complex, Peshawar:** Laila Khadim**, Zahid Aman**, Muhammad Aasim, Muhammad Waqar, Iqra Zaman, Muhammad Sheraz Ali, Mahnoor Musharaf, Hafsa Khan, Sadia Ambreen, Aqdas Faiz, Aiman Haroon, Mehreen Mushtaq Ahmad, Alishba Fatima, Maham Mehmood, Eeman Shehzad, Iman Ali, Shandana Gul, Maimoona Naeem.

**Holy Family Hospital, Rawalpindi:** Shanza Abbasi**, Jahangir Sarwar Khan**, Rubina Shahzad, Gohar Rasheed, Ashraf, Usman Qureshi, Lubna Ijaz, Zubair.

**Isra University Hospital, Hyderabad:** Fatima Siddiqui**, Juwereya Memon**, Farhana Anjum, Mohammad Shahid Khan, Adeena Noor, Fiza Lashari, Alizeh Fatima Memon, Mariam Qureshi, Amna Gopang, Aisha Shah, Marvi Mirbhar, Anousha Afzal, Abdullah Memon, Seerat Hameem, Sadia Hameed Lashary, Khushboo Devi, Adeel Rehman, Ahmed Mustafa Burney.

**Jinnah Hospital, Lahore:** Muhammad Alyaan Arshad**, Shahid Mehmood**, Muhammad Umer Farooq, Raffay Ali Gillani, Nabeel Chodhary, Saima Iqbal, Laveeza Fatima, Minahil Iqbal, Faseeh Fatima, Sara Khan, Anzil Adnan, Farooq Ahmad, Rubab Zahra, Sehar Fatima, Muhammad Fazeel Shahid, Muhammad Zulkifl, Hadia Nadeem, Muhammad Subhan Saleem, Hassan Mehmood, Muhammad Ahrar Bin Naeem, Tooba Nihal, Ayesha Yousaf Khan, Yumna Naeem, Aqsa Zainab, Laiba Ali, Saad Masood, Ali Azlan, Abdul Wasay, Abdul Wahab Mirza, Marium Mansoor, Maryam Baloch, Zain Ali Nadeem, Maha Malik, Muhammad Rafay Paracha, Muhammad Hamza Awais Khalid, Hammad Jehangir, Yashfeen Amjad, Shahab Zafar, Sameen Shafqat, Haider Ashfaq, Hamza Ashraf, Sophia Ahmed, Aaila Hameed, Areej Iftikhar, Asna Moghis, Umar Akram, Faryal Khan, Samra Shafique, Muhammad Sohaib Khan, Fasih Khalil Ur Rehman, Abdullah Arshad.

**Khyber Teaching Hospital, Peshawar:** Ali Nawaz Khan**, Muhammad Zareen**, Muhammad Idris Khan, Fauzia Afridi, Sadaf Faryal, Arif Hussain, Romana Bibi, Sara Jamil, Waseem Jamil.

**Lady Reading Hospital, Peshawar:** Sundas Mehreen**, Abdul Wahab**, Haseena Rehman, Saima Khattak, Faaiz Ali Shah, Ilyas Khan, Ijaz Ahmad, Kainat Ullah Khan, Ume Kulsum, Tariq Shah, Abdullah Khan, Babur Farid, Abbas Bangash.

**Lahore General Hospital, Lahore:** Syed Mubeen Ahmed**, Ahmed Naeem**, Yaser Ul Din Hoti, Farooq Afzal, Adeel Hamid, Najam Gohar, Mahin Qudeer Sheikh, Umar Abdullah, Waleed Anjum, Uzair Ahmad, Abdullah Masood, Aroob Farooqi.

**Liaquat University Hospital, Jamshoro:** Suman Memon**, Vashdev Khimani**, Ahmed Hussain Pathan, Qurat Ul Ain, Fahad Shabir, Taskeen Waheed, Usama Ahmed Qureshi, Ramsha Anwar, Nadia Sadiq Memon.

**Lyari General Hospital, Karachi:** Sanjeet Kumar**, Farhat Bano**, Javeria Farooq, Faisal Aftab, Fehmina Raza, Muhammad Shaharyar Sabeeh.

**Nishtar Medical Hospital, Multan:** Nouman Arif**, Shams Bukhari**, Aamir Shahzad, Rizwan Sharif, Munazza Khalid, Muhammad Ali, Ali Waqas, Syed Fouz Hyder Abidi, Muhammad Uzair Rafique, Musfirah Younis, Wajeeha Younas, Rimsha Afzal, Laraib Mubashir, Abdul Rafay, Aqsa Bilal, Naima Khalid.

**Northwest General Hospital, Peshawar:** Mohammad Nouman Arshad**, Almas Fasih Khattak**, Tarbia Hamid, Syed Sarmad Bukhari, Amjad Ali Shah, Saifullah Shafiq, Muhammad Hamza, Fatima Khan

**Pakistan Railway Hospital, Rawalpindi:** Aamena Akhtar**, Kiran Rehman, Ayesha Mobeen, Masooma Husnain, Zainab Zia, Iqra Nasir, Mohad Bin Asghar, Syed Usman Husain Shah, Zobia Majeed, Lareb Basharat, Sumaira Yasmeen, Raja Adnan, Rehan Ahmed Khan, Sohail Iqbal Sheikh, Sameen Rehan.

**Peoples University of Medical & Health Sciences for Women, Nawabshah:** Sobul Khan**, Mohammad Ali Sohail**, Naeem Karim Bhatti, Abdul Razaque Mari, Manahil Iftekhar, Sanish Abassi, Wardah Nizamani, Amna Suhail, Mahek Gul, Summiya Bhatti, Kiran Manwani.

**Punjab Institute of Neurosciences, Lahore:** Haseeb Mehmood Qadri**, Asif Bashir**.

**Rai Medical College Teaching Hospital, Sargodha:** Areesha Fazil Awan**, Rai Muhammad Aslam**, Tabassum Firdous.
